# Inhibition by stabilization: targeting the *Plasmodium falciparum* aldolase–TRAP complex

**DOI:** 10.1186/s12936-015-0834-9

**Published:** 2015-08-20

**Authors:** Sondra Maureen Nemetski, Timothy J Cardozo, Gundula Bosch, Ryan Weltzer, Kevin O’Malley, Ijeoma Ejigiri, Kota Arun Kumar, Carlos A Buscaglia, Victor Nussenzweig, Photini Sinnis, Jelena Levitskaya, Jürgen Bosch

**Affiliations:** Department of Biochemistry and Molecular Pharmacology, New York University School of Medicine, New York, USA; Institute for Systems Genetics, New York University School of Medicine, New York, USA; Department of Molecular Microbiology and Immunology, Johns Hopkins University Bloomberg School of Public Health, Baltimore, USA; Department of Biochemistry and Molecular Biology, Johns Hopkins University Bloomberg School of Public Health, Baltimore, USA; Department of Medical Parasitology, New York University School of Medicine, New York, USA; Michael Heidelberg Division of Pathology of Infectious Diseases, Department of Pathology, New York University School of Medicine, New York, USA; Instituto de Investigaciones Biotecnológicas-Instituto Tecnológico de Chascomús (IIB-INTECH), Universidad Nacional de General San Martín-CONICET, 1650 San Martín, Buenos Aires Argentina; Johns Hopkins Malaria Research Institute (JHMRI), Baltimore, USA; Department of Pediatrics, Phyllis and David Komansky Center for Children’s Health, New York-Presbyterian Hospital-Weill Cornell Medical College, New York, USA; Department of Animal Sciences, School of Life Sciences, University of Hyderabad, Hyderabad, 500046 India

**Keywords:** *Plasmodium falciparum*, Glideosome, Drug discovery, Protein–protein interaction, Inhibitor, Stabilizer, Virtual library screening, X-ray crystal structure, Malaria

## Abstract

**Background:**

Emerging resistance of the malaria parasite *Plasmodium* to current therapies underscores the critical importance of exploring novel strategies for disease eradication. *Plasmodium* species are obligate intracellular protozoan parasites. They rely on an unusual form of substrate-dependent motility for their migration on and across host-cell membranes and for host cell invasion. This peculiar motility mechanism is driven by the ‘glideosome’, an actin–myosin associated, macromolecular complex anchored to the inner membrane complex of the parasite. Myosin A, actin, aldolase, and thrombospondin-related anonymous protein (TRAP) constitute the molecular core of the glideosome in the sporozoite, the mosquito stage that brings the infection into mammals.

**Methods:**

Virtual library screening of a large compound library against the *Pf*Aldolase–TRAP complex was used to identify candidate compounds that stabilize and prevent the disassembly of the glideosome. The mechanism of these compounds was confirmed by biochemical, biophysical and parasitological methods.

**Results:**

A novel inhibitory effect on the parasite was achieved by stabilizing a protein–protein interaction within the glideosome components. Compound 24 disrupts the gliding and invasive capabilities of *Plasmodium* parasites in in vitro parasite assays. A high-resolution, ternary X-ray crystal structure of *Pf*Aldolase–TRAP in complex with compound 24 confirms the mode of interaction and serves as a platform for future ligand optimization.

**Conclusion:**

This proof-of-concept study presents a novel approach to anti-malarial drug discovery and design. By strengthening a protein–protein interaction within the parasite, an avenue towards inhibiting a previously “undruggable” target is revealed and the motility motor responsible for successful invasion of host cells is rendered inactive. This study provides new insights into the malaria parasite cell invasion machinery and convincingly demonstrates that liver cell invasion is dramatically reduced by 95 % in the presence of the small molecule stabilizer compound 24.

**Electronic supplementary material:**

The online version of this article (doi:10.1186/s12936-015-0834-9) contains supplementary material, which is available to authorized users.

## Background

Despite recent advances in treatment and prevention, malarial disease continues to afflict hundreds of millions of people every year, with growing resistance to current therapies [1–5]. Innovative treatments targeting hitherto under-exploited aspects of plasmodial biology are needed.

*Plasmodium*, as with other protozoan parasites belonging to the phylum *Apicomplexa*, progress through their life cycle by invading host cells. Gliding and active host cell invasion are thus crucial for these organisms, and are facilitated through an actin/myosin motor complex located beneath the parasite’s plasma membrane [6–8]. Herewith, the bridging enzyme *Pf*Aldolase, which binds actin in addition to its role in glycolysis [9], plays a key role: it connects the actin/myosin motor to trans-membrane adhesins of the thrombospondin-related anonymous protein (TRAP) family, which are expressed in a life-cycle stage specific manner [10]. Thus, during plasmodial liver, blood and transmission stages, *Pf*Aldolase binds the conserved C-termini of the plasmodial paralogs TRAP, MTRAP and CTRP, respectively [10–15] as well as other interaction partners such as the cytoplasmic tail of AMA-1 [16].

**Fig. 1:**
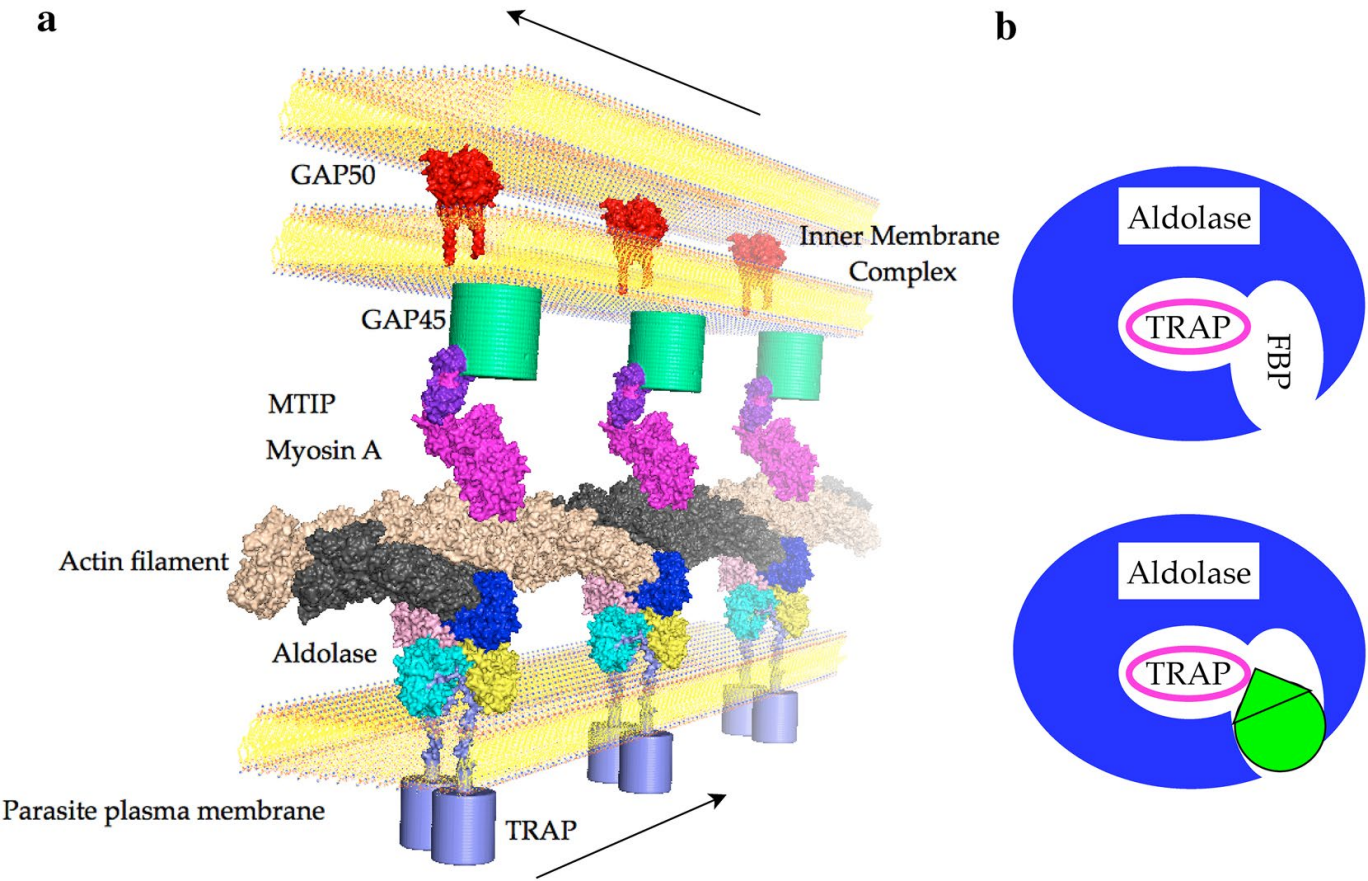
Schematic overview of the glideosome components and principle of enzyme inhibition and complex stabilization. **a** The gliding machinery is anchored between the parasite plasma membrane and the inner membrane complex (IMC). Aldolase mediates a bridging function between short actin filaments and TRAP. **b** Simplified model of a single aldolase sub-unit with TRAP and F16P binding sites. TRAP-binding promoters (*green*) occlude access to the catalytic site of aldolase.

During the gliding and invasion processes, TRAP molecules are translocated from the anterior to the posterior end of the parasite, where they are cleaved within their transmembrane domain by a rhomboid protease [17, 18]. The cleavage reaction leaves the extracellular TRAP domains bound to the host cell or substrate, while the cytoplasmic C-termini, also referred to as TRAP-tails, remain bound to *Pf*Aldolase, from which they are believed to dissociate and be recycled for degradation over time [18, 19]. Un-liganded *Pf*Aldolase molecules are then available for binding other TRAP molecules, thus enabling continuous gliding motion on the host cell surface, which constitutes a necessary prerequisite for subsequent invasion [20]. A schematic overview of the glideosome components that were identified via combinatorial pull-down experiments in *Plasmodium* parasites [10] is given in Fig. 1a.

The study presented here aims to inhibit parasite motility and infectivity by targeting the aldolase–TRAP interaction within the glideosome. As an enzyme of the glycolytic pathway, however, aldolase is well conserved throughout all kingdoms (204/365 identical residues between human and *Plasmodium* aldolase, see Additional file 1). Inhibitor design targeting the *Pf*Aldolase molecule and its binding partners must therefore meet the challenge of avoiding cross-reactivity with human aldolase enzymes. This study employs a novel approach to rational drug design to meet this challenge.

While traditional, targeted inhibitor-design approaches are usually geared towards finding small molecules that prevent protein–protein interactions (PPI) critical to the pathogen [21], the hypothesis explored here is counter-intuitive: strengthening, instead of hindering, the *Pf*Aldolase–TRAP interaction is hypothesized to inhibit motility and invasion. The rationale for this counterintuitive approach is that the hybrid molecular surface formed partly by *Pf*Aldolase and partly by TRAP in the bound state of the complex is unique to the parasite and could allow *Plasmodium*-specific targeting with small molecules that would not cross-react with the human orthologues (Fig. 1b). More importantly, a fast dissociation of bound TRAP-tails (after protease cleavage) is critical for *Plasmodium*’*s* ability to recycle *Pf*Aldolase molecules during gliding motility and host cell invasion [17, 18]. Therefore, a small molecule designed to strengthen the *Pf*Aldolase–TRAP interaction should render this crucial dissociation process slow and inefficient, thus leading to an imbalance of this dynamic system and concomitant locomotion defects and likely reduced cell invasion. Equally importantly, the ternary complex of *Pf*Aldolase–TRAP and a stabilizing agent would be expected to interfere with glycolytic activity as the active site is occluded. It is unknown if the liver stage parasite relies on glycolytic activity for energy generation as does the blood stage form of the parasite [22]. However, *Pf*Aldolase is constitutively expressed in blood and gametocytes stages, suggesting it may be expressed, and is likely required, during liver stages as well (PlasmoDB PF3D7_1444800). A similar approach to stabilizing a PPI is described by Mecozzi et al. where they identified small molecules by virtual screening that were capable of stabilizing the Vps35–Vps26 interaction [23].

This hypothesis was tested using biophysical and biochemical assays as well as in vitro culture experiments with *Plasmodium falciparum* and *Plasmodium berghei* parasites to demonstrate that small molecules identified by virtual library screening (VLS) show an effect on gliding motility and hepatocyte invasion. A primary screen, which was comprised of VLS, *Pf*Aldolase catalytic activity, and thermal stability in the presence of small molecules, identified several compounds active in two or more assays. These were then further validated in two parasite specific assays, one investigating the impact on gliding motility and the second testing if parasites treated with small molecules are hindered in invasion of liver cells. Finally, the ternary co-crystal structure of *Pf*Aldolase–TRAP with compound 24 stabilizing the interaction was determined. The ternary complex is observed in all four copies of the *Pf*Aldolase tetramer represented in the crystal structure.

## Methods

### Compound library and chemicals

The screening library of 315,102 chemicals was provided as a structure description file (SDF) from the Chembridge Corporation (San Diego, CA, USA), as previously described. Unless otherwise noted, all compounds used in the in vitro and in vivo assays were obtained in powdered form from the Chembridge Corporation, and initially dissolved in 100 % DMSO to obtain 100 mM stock solutions, which were stored at 4 °C or −20 °C. Whenever possible, working dilutions in the relevant buffers were made within 24 h of the experiments in which they were used.

### Computational methods

All computational work, including receptor modelling, VLS, docking, and hit-list post-processing was completed using tools in the ICM software suite produced by Molsoft, LLC (Version 3.7, La Jolla, CA, USA) with default parameters. VLS was performed as previously described [24]. The PocketFinder function of ICM was used to render solvent pockets suitable for small molecule ligand binding on the molecular surface of 3D structural protein models as previously described [25]. The crystal structure of the *Pf*Aldolase TRAP complex present in PDB ID 2pc4 was used as the starting point for all of the modelling, VLS and docking described in this study.

### Expression and purification of *Plasmodium falciparum* aldolase

Cloning, expression, and purification of *P. falciparum* aldolase in *Escherichia coli* was performed using either of two previously described methods [26]. Prior to catalysis assays, the GST-tag was removed from the tagged protein using the Novagen Factor Xa Cleavage-Capture Kit according to manufacturer’s instructions (EMD Biosciences, San Diego, CA, USA).

### Synthetic peptides

Synthetic peptides derived from the cytoplasmic tails of *P. falciparum* and *P. berghei* TRAP were custom-synthesized by Genemed Synthesis, Inc (TX, USA). These included *Pf*TRAP25 (ETLGEEDKDLDEPEQFRLPEENEWN), *Pf*TRAP6 (EENEWN), *Pb*TRAP25 (VMADDEKGIVEDEGFKLPEDNDWN), and *Pb*TRAP6 (EDNDWN).

### Thermal shift assay

The results reported here measured the effect of the VLS hits on a complex of recombinant *P. falciparum* aldolase and the *Pb*TRAP6 peptide described above. The assays were conducted and analysed as per previously published protocols in triplicates [27, 28].

### Aldolase catalysis assay

The protocol utilized here was based on that provided by Sigma-Aldrich^®^ (St. Louis, MO, USA), and all of the reagents (listed below) other than drugs, TRAP and aldolase were obtained from that company as well. Briefly, aldolase was pre-incubated for 10 min, +/− TRAP peptide (*Pf*TRAP25, described above), +/− compound or DMSO at room temperature. The other reagents (α-GDH/TPI, β-NADH, F16P in order) were then added, yielding a final reaction mixture containing 0.02 units/ml aldolase (1 unit = amount of aldolase required to convert 1 μM of F16P to DHAP and G3P per minute at pH 7.4 and 25 °C; for these studies, this usually amounted to ~50 nM aldolase, based on an estimated purification yield of 10 units/mg aldolase), 2 mM F16P, 0.13 mM β-NADH, 2 units/ml α-GDH/TPI (1 unit = amount of αGDH required to convert 1.0 μM of DHAP to α-glycerophosphate per min at pH 7.4 and 25 °C), 100 nM TRAP (or DMSO), and 5–100 μM compound (or DMSO) in catalysis buffer (0.2 M glycine titrated to pH 7.3 with Trizma Base). A buffer with low ionic strength was used to avoid interference with the electrostatic interactions between aldolase and TRAP. Reactions were carried out either in a final volume of 1 ml, in standard plastic cuvettes (assays with *Pf*TRAP25), or in a 96-well format with 100 µl reaction volume (assays with *Pb*TRAP6). NADH consumption was measured at 340 nm for 10 min at 25 °C, using a SpectraMax M2e Microplate Reader (Molecular Devices, Sunnyvale, CA, USA). As many of the compounds had measurable inherent absorbance at 340 nm, the baseline absorbance of each compound when dissolved in catalysis buffer at the tested concentration was measured and subtracted from the values obtained during the kinetic run. Suramin, a known aldolase inhibitor, was used as a positive control for aldolase inhibition [29].

To test if compound 24 had an inhibitory effect on human aldolase, rabbit muscle aldolase was used, which is 99.3 % sequence identical to human aldolase. The assay was performed as described previously, however in the absence of TRAP-peptide to identify if the compound inhibited catalytic activity by itself.

### X-ray crystallography

Compounds were co-crystallized with the *Pb*TRAP6 peptide and purified *P. falciparum* aldolase as per previously published protocols [26]. Crystallization trials of *Pf*Aldolase with TRAP in the presence of different concentrations of the small molecules were set up at 20 °C. In most cases crystals of different sizes appeared within 1 week, many of the small molecules resulted in precipitated solutions at higher concentrations. Multiple synchrotron datasets were collected from crystals grown in the presence of 2 mM compounds 1, 2, 3, 5, 6, 8, 11, 12, 13, 21, 24, 30, 42, 43, 49, and 54, resulting in diffraction from 2.1 to 4 Å resolution. Data processing and scaling was carried out with XDS/XSCALE [30]. The scaled data were then subjected to molecular replacement with the coordinates of 2pc4 [26] followed by automatic refinement using the Phenix suite [31]. All coordinates were refined below an R_work_/R_free_ of 28/32 prior to inspection of the electron density maps. Every dataset was visually inspected using Coot [32] and small molecule ligands were searched for either automatically using the find unmodelled blob function or manually by inspecting the four TRAP binding sites of each aldolase subunit near residues R48 and R309. The ternary complex of *Pf*Aldolase–TRAP with compound 24 was refined to an R_work_/R_free_ of 18.7/24.8 with one Ramachandran outlier as reported by Molprobity [33]. Compound 24 ligand occupancy was automatically refined using Phenix refine to 0.81–0.87, indicating a high occupancy of the compound in the four binding sites. A summary of the data reduction and refinement statistics are provided in Table 1. The structure factors and coordinates of the final model have been deposited with the PDB under accession code 4TR9.

**Table 1:** Data collection and refinement statistics for PDB entry 4TR9

Beam line	SSRL 12-2, micro focus
Wavelength (Å)	0.9795
Resolution range (Å)	44.85–2.11 (2.19–2.11)
Space group	P 2_1_ 2_1_ 2_1_
Unit cell (Å, °)	69.89 139.56 142.10 90 90 90
Total reflections	281,841 (1,566)
Unique reflections	49,244 (642)
Multiplicity	5.7 (2.4)
Completeness (%)	61.09 (8.08)
Mean I/sigma (I)	21.47 (1.04)
Wilson B-factor	36.99
R-merge	0.0702 (0.91)
R-meas	0.077
CC1/2	0.999 (0.654)
CC*	1 (0.889)
R-work	0.187 (0.282)
R-free	0.248 (0.289)
Number of non-hydrogen atoms	11,395
Macromolecules	10,889
Ligands	84
Water	422
Protein residues	1,416
RMS (bonds)	0.010
RMS (angles)	1.38
Ramachandran favoured (%)	94
Ramachandran outliers (%)	1
Clashscore	8.58
Average B-factor	46.50
Macromolecules	46.30
Ligands	59.70
Solvent	48.30

Statistics for the highest-resolution shell are shown in parentheses.

### Surface plasmon resonance assay

A CM5 chip was prepared and conjugated with Neutravidin, allowing the subsequent capturing of biotinylated peptides, as described in [34]. All experiments were carried out at 25 °C using a running buffer consisting of 10 mM Hepes pH 7.5, 150 mM NaCl, 1 mM MgCl_2_, 0.2 % Tween 20, 1 % DMSO. Purified *Pf*Aldolase was passed over a reference flow cell as well as over a *Pf*TRAP-, *Pv*TRAP- and *Pf*MTRAP-tail exposing surface. Binding of *Pf*Aldolase was measured in the presence of different concentrations ranging from 125 to 1,000 µM of compound 24. All measurements were performed in triplicates interspersed by blank injections. Data analysis was carried out with Scrubber (BioLogic Software) using double referencing method and correcting for DMSO absorption effects.

### Hepatocyte viability assay

The VLS hits were screened for their affects on cultured HC-04 hepatocytes (ATCC, Manassas, VA, USA) as previously described [35–37]. To determine toxicity of compounds 1, 3, 24, 42 and 43 on human hepatocytes, human hepatocyte cell line HC-04 capable of supporting *P. falciparum* development in vitro [38] was exposed to 1 mM of each compound for 96 h followed by Annexin V-APC and Propidium Iodide staining done according to manufacturer’s instructions (Apoptosis Detection Kit, eBioscience Inc, San Diego, CA, USA). Samples were analysed using flow cytometry (FACS-Scan, BD Biosciences) and the percentage of Annexin V negative/Propidium Iodide negative viable cells was calculated using FlowJo analysis software (Tree Star Inc, Ashland, OR, USA).

### Sporozoite motility assay

Compounds were tested for their effect on *P. berghei* sporozoite motility using established protocols [39, 40] For the assays described here, sporozoites were pre-incubated with each compound at 500 μM for 10 min at 28 °C and the sporozoites remained in the presence of the compound (or DMSO) during the 1 h-long assay at 37 °C. The quantity of motile parasites, and the numbers of their trails were then calculated to assess the compounds’ effects.

### Sporozoite invasion assay

The sporozoite neutralization assay was carried out as previously described [41]. Briefly, *P. berghei* sporozoites were pre-incubated with 500 μM of the drugs or DMSO, and then allowed to infect human HepG2 cells (ATCC Collection). The HepG2 cells were collected after 40 h, and the infectivity of the parasites was quantified by real-time PCR using primers specific for the *P. berghei* 18S rRNA [42].

## Results

### Identification of ligand-accessible pockets through VLS

**Fig. 2:**
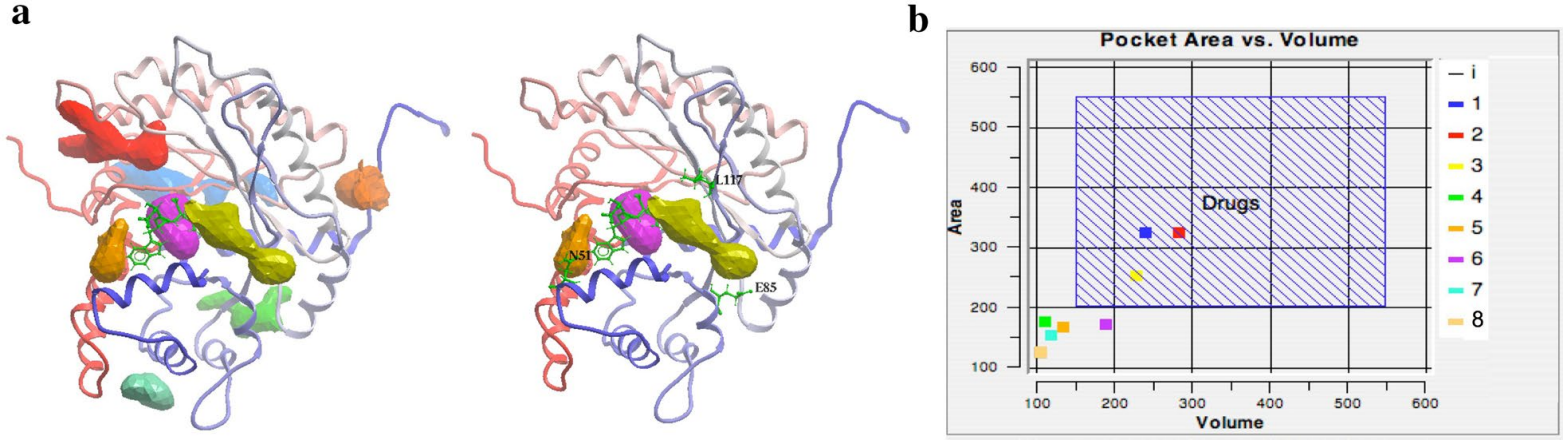
Overview of the *Pf*Aldolase–TRAP druggable pockets. **a** Potential ligand-binding pockets in *Pf*Aldolase. Eight pockets were identified in the cocrystal structure of aldolase bound to TRAP (PDB ID: 2pc4 [26]). Aldolase is shown as a *ribbon coloured* in a smooth gradient from *blue* at its N-terminus to *red* at its C-terminus. TRAP is shown as a *green* *ball-and-stick* model. Only pockets 3, 6 and 8 are displayed because these three were targeted by the design with VLS since they are contacted by three non-conserved residues within aldolase—N51, E85, L117 (*green balls-and-sticks*)—as well as by the TRAP peptide. **b** Plot of the surface area (Å^2^) vs volume (Å^3^) for the eight pockets. Pockets 1, 2 and 3 fall within the 100–500 Å^2/3^ area/volume range, which is the usual area/volume for most FDA-approved drugs, following Lipinski’s rule of five [70].

Small molecules with the potential to stabilize the interaction of the hybrid interface of TRAP with *Pf*Aldolase were identified by VLS on a previously described co-crystal structure of *P. falciparum* aldolase in complex with a short cytoplasmic tail of TRAP [26] (Fig. 1b). A necessary feature for targeting a particular molecular surface with VLS is the presence of an optimally located, appropriately sized ‘druggable pocket’ (i.e., a ligand-accessible cavity or surface) against which to screen a chemical library [43]. Suitable pockets in the *Pf*Aldolase–TRAP target (PDB code 2pc4, 2.4 Å resolution, R_work_/R_free_ = 20.1/25.0) [26] were located with the ICM PocketFinder [25] (Molsoft, LLC., La Jolla, CA, USA), setting pocket form and identity as key strategic parameters. For the present experimental design, the pocket walls needed to be formed by the mixed surface of both TRAP and *Pf*Aldolase (Fig. 1b). As shown in Fig. 2a, b and Additional file 1, three pockets (coloured yellow, purple, and orange, respectively) met this criteria. These pockets juxtapose with the TRAP binding site, contacting the non-conserved *Pf*Aldolase residues, N51, E85 and L117, as well as TRAP itself (balls-and-sticks in the Figure). While two of the pockets (purple and orange in Fig. 2a, b) individually have smaller-than-desirable area-to-volume ratios for drug binding [43], compound fragments fitting them could still be useful for a fragment-based approach to the design of the sought-after drug. Additionally, their key locations within the target region justified including them in the target VLS area.

### Target site modelling

The precise conformation of the target pocket may strongly influence the selection of compounds by the VLS algorithm, as the target site is not flexible during the docking procedure. Therefore, in addition to screening the co-crystal structure of *P. falciparum* aldolase bound to a hexapeptide derived from the C-terminus of *P. berghei* TRAP6 (PDB ID 2pc4, ‘2pc4 model’), additional screens were carried out against two additional models of the complex generated in silico: one in which the *P. berghei* TRAP sequence (EDNDWN) was modified to its *P. falciparum* counterpart (EENEWN, ‘falciparum model’), and one in which the final TRAP residue was modified to alanine (EENDWA), in order to simulate induced fit via the ‘gapped-pocket’ method (‘gapped-pocket model’) [44]. The different VLS receptor models and the areas in which the docking was concentrated are shown in Additional files 2 and 3.

### Virtual hit group selection through target site docking

315,102 small molecules, representing a sub-set of the ChemBridge^®^ hit2lead database (San Diego, CA, USA), were docked to the three different conformations of the target site using the ICM-VLS algorithm (Molsoft LLC, La Jolla, CA, USA). Three independent virtual screens against each receptor model, specifically targeting the *Pf*Aldolase–TRAP interface and surrounding residues, yielded 182 unique hits. To further narrow this preselection, these 182 compounds were re-docked to their respective receptors using the slower, more energetically accurate ICM-DOCK algorithm (Molsoft LLC, La Jolla, CA, USA). This step eliminated hits whose re-docked poses and/or energy scores differed significantly from the initial VLS results, as well as those with predicted lipophilicity (partition coefficients; cLogP) <−2 or >4. 60 of the 69 remaining compounds were then purchased from ChemBridge (San Diego, CA, USA) for in vitro and cell-based assay testing. The individual small molecule structures as well as details of the VLS and docking results for these 60 compounds are listed in Additional file 4.

### In vitro hit validation by enzymatic activity and thermal stability assays

In keeping with the novel hypothesis presented here, the initial 60 docking hits were subjected to biochemical and biophysical tests to identify those small molecules that would actually enhance the *Pf*Aldolase–TRAP interaction and thereby occlude access of fructose 1,6-bisphosphate as a substrate to the active site of the enzyme (Fig. 1b). As mentioned above, TRAP binding has an inhibitory effect on aldolase’s glycolytic activity as previously demonstrated [26] (Fig. 1b).

**Fig. 3:**
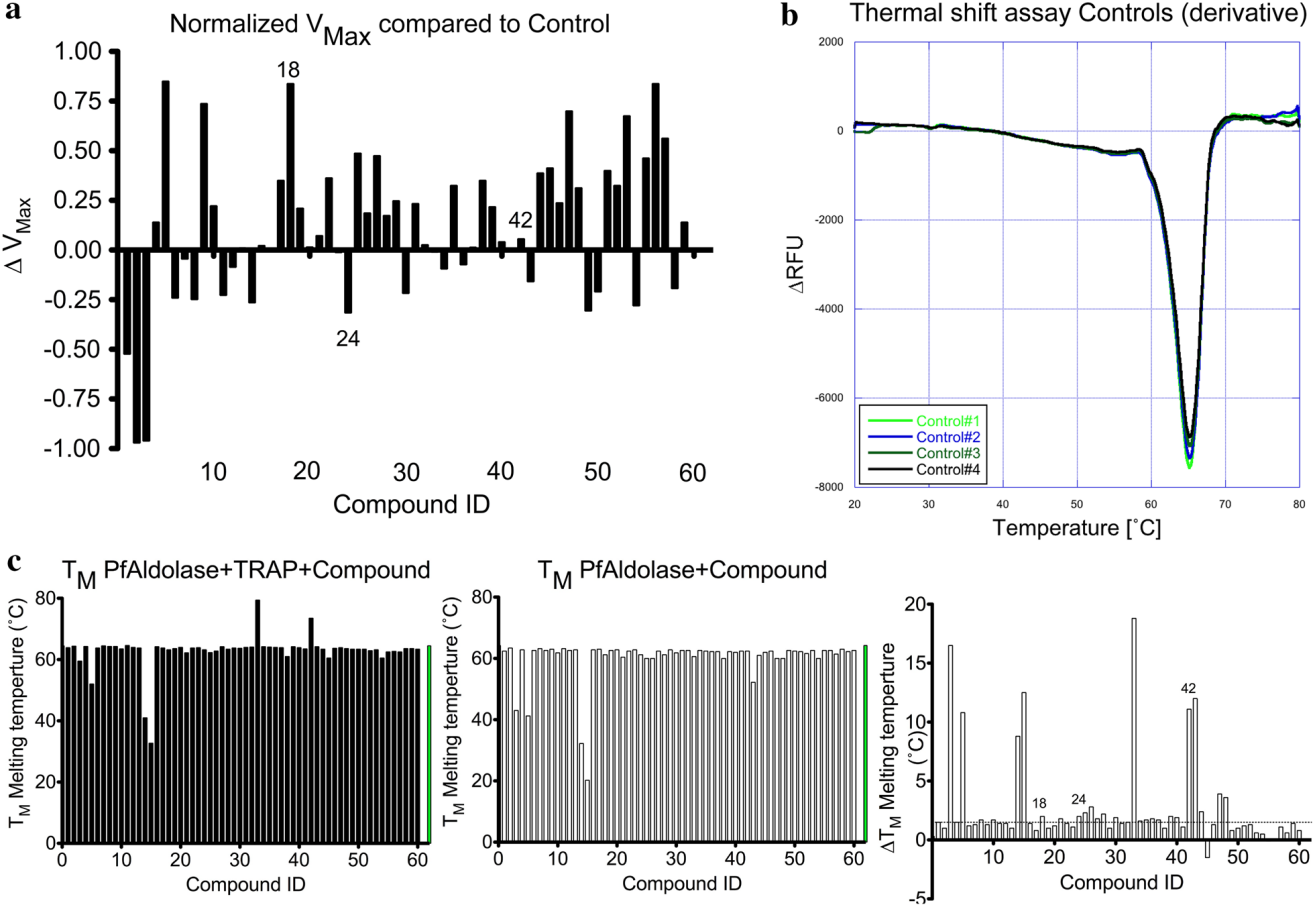
*Pf*Aldolase catalytic activity and thermal stability in the presence of compounds. **a** Normalized *bar graph* representing the change in ∆V_max_ relative to the control in the presence of each of the 60 ordered compounds. All assays were carried out in the presence of TRAP as used for the reference control. A negative ∆V_max_ indicates further inhibition of glycolysis in the presence of the compound compared to *Pf*Aldolase with TRAP only. A positive ∆V_max_ indicates displacement of TRAP by the compound resulting in an increased catalytic activity. Compounds inhibiting invasion in an in vitro culture assay are *numbered* in the plot (see Fig. 5). **b** Thermal stability assay derivative curves of four controls of *Pf*Aldolase with *Pf*TRAP. The T_M_ is indicted by the minima of the derivative at approximately 65 °C. **c** Average of a triplicate thermal stability assay in the presence of small molecules with and without *Pf*TRAP peptide. The *green bar* represents the melting temperature of the control without compound. The change in thermal stability (∆T_M_) in the presence of *Pf*TRAP per compound is depicted in the last graph. The *dotted line* represents the cutoff of a shift greater than 1.5 °C.

To this end, the effects of the 60 pre-selected VLS hit compounds on *Pf*Aldolase’s enzyme activity were first investigated in the presence and absence of TRAP. TRAP is a competitive inhibitor of *Pf*Aldolase as the binding sites of the glycolytic substrate fructose 1,6-bisphosphate (F16BP) and TRAP partially overlap [26]. Following the method of Döbeli et al. [22], *Pf*Aldolase enzyme activity was assayed by monitoring NADH consumption. The presence of the inhibitor TRAP expectedly caused a dose-dependent decrease in NADH consumption as previously published [26]. Hit compounds were considered potential stabilizers of the *Pf*Aldolase–TRAP interaction if they promoted an additional decrease in NADH consumption/*Pf*Aldolase activity in the presence *versus* absence of TRAP. The change in the V_max_-rate compared to the attenuated *Pf*Aldolase–TRAP control distinguishes between inhibitory molecules with a negative ∆V_max_ and TRAP-displacing molecules with a positive ∆V_max_ (Fig. 3a). TRAP-displacing molecules would likely interfere with human aldolase as the residues to which TRAP binds are identical between human and *Plasmodium* aldolase and are therefore undesired hits [26]. In order to identify sequence-specific differences in compound binding, all 60 VLS hits were assayed independently against *Pf*Aldolase with peptides derived from either *P. berghei* (DWA) or *P. falciparum* TRAP (DWN).

Furthermore, the thermal stability [27] of the *Pf*Aldolase–TRAP complex was investigated with or without enzyme inhibition promoting hit compounds, in order to exclude effects resulting merely from structural destabilization due to compound addition. The melting temperature (T_M_) of the *Pf*Aldolase–TRAP complex was assayed in the presence versus absence of hit compounds, where a strongly negative T_M_-shift would indicate a destabilizing, denaturing effect. However, compounds causing only marginally negative T_M_’s or positive T_M_’s shifts compared to the *Pf*Aldolase–TRAP control were considered as potentially viable hits for further analysis (Fig. 3b, c).

At 100 μM final concentration of the compounds, 13 (compounds 3, 5, 14, 15, 18, 25, 26, 28, 33, 42, 43, 47, and 48) produced a positive shift of >2 °C in the melting point of a complex of recombinant *P. falciparum* aldolase and the same *P. berghei* TRAP hexapeptide used to solve the TRAP–aldolase co-crystal structure, suggesting a stabilizing effect (Fig. 3b, c). Interestingly, five compounds produced a negative T_M_ shift of >2 °C compared to the control (compounds 3, 5, 14, 15, 43) when no TRAP-peptide was present, some of which could then be stabilized when TRAP was added (Fig. 3c, third panel).

### Preliminary ligand based SAR analysis after primary screen

**Fig. 4:**
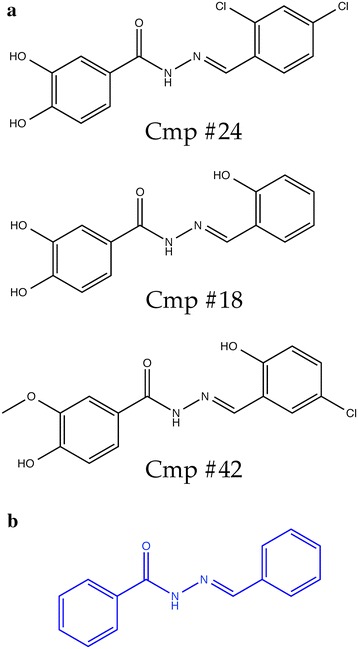
Identified scaffold and chemical formulas. **a** Chemical formulas of compounds 18, 24 and 42 showing a phenotypic effect on parasite gliding and parasite invasion of liver cells. **b** Frequently observed common scaffold of our VLS hits was a *N*-(benzylideneamino)benzamide ring system.

Many of the VLS hits share similar chemical scaffolds (Fig. 4; Additional file 4). In particular, many of the compounds that were active in the catalysis or thermal shift assays, including compounds 1, 9, 13, 16, 17, 19, and 21, as well as compounds 18, 24 and 42, which were also active in in vitro motility and cell invasion assays (see below), contained *N*-(benzylideneamino)benzamide (Fig. 4b). The docking results and the crystallographic data for the active compounds suggest that several of these hits also make similar hydrogen-bonding and electrostatic contacts with the *Pf*Aldolase–TRAP complex via functional groups extending off of the scaffold’s two benzene rings (Additional file 5).

### In vitro parasite hit validation by gliding motility and liver cell invasion assays

**Fig. 5:**
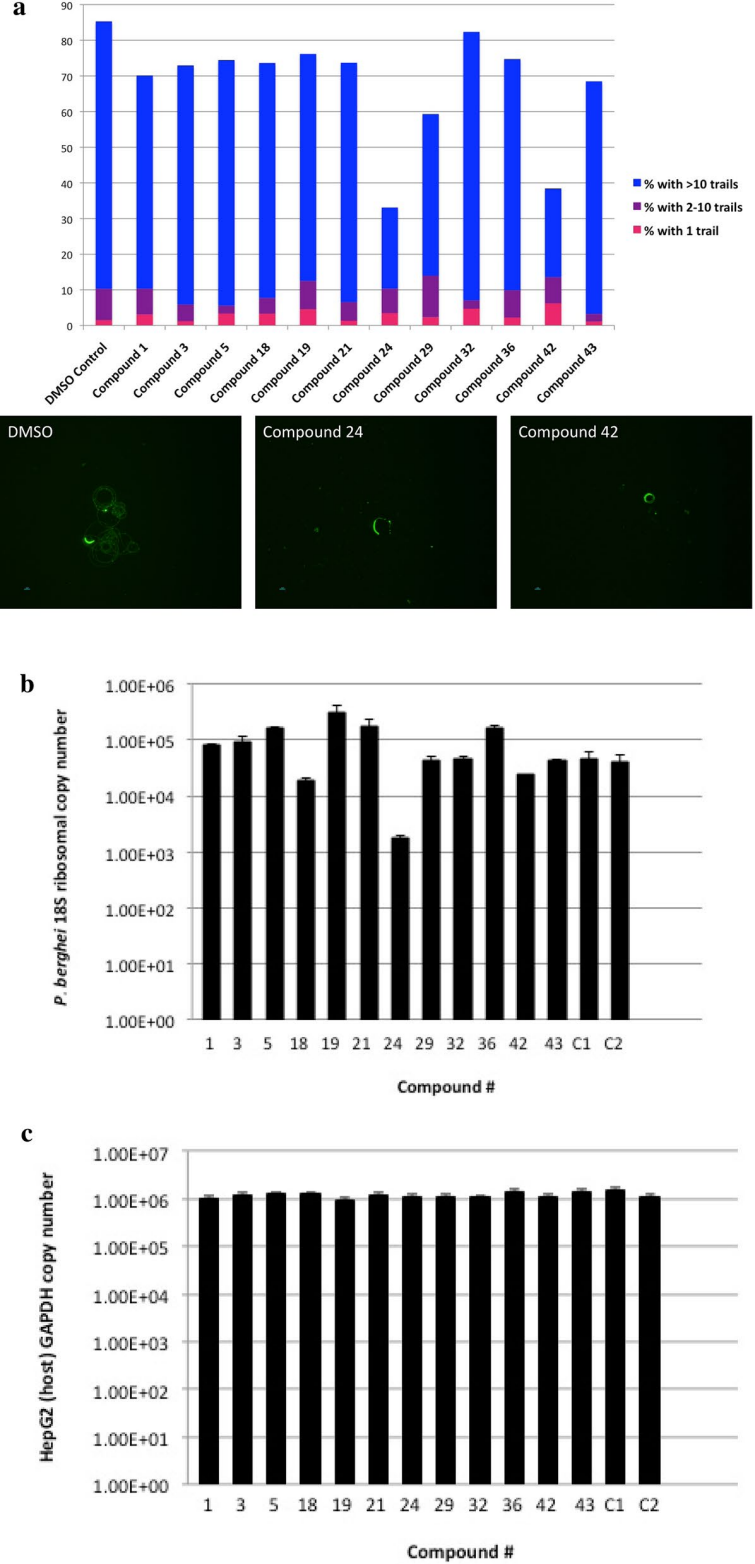
Gliding motility and hepatocyte invasion assay in the presence of compounds. **a** Compounds 24 and 42 impair the ability of *P. berghei* sporozoites to glide on glass coverslips. The total height of *bars* represent the percentage of sporozoites that were motile (produced one or more trails on a glass slide) during the assay period. Untreated sporozoites produce >10 trails under the assay conditions. As shown here, sporozoites treated with compounds 24 or 42 were less motile overall and produced fewer trails than the DMSO controls. In this experiment, treatment with DMSO, compound 24, or compound 42 produced 85, 33, and 38 % motile sporozoites, respectively. Fluorescent microscopy images of a representative sporozoite (*green crescent*) and its gliding path (*green spirals*) for three assay treatments: DMSO control (*left*), compound 24 (*centre*), and compound 42 (*right*). Sporozoite trails were visualized using a biotinylated antibody to CSP. While some sporozoites treated with compounds 24 or 42 did produce >10 trails, most of them produced no or few trails as shown here. **b** HepG2 liver invasion assay in the presence of small molecules. Compounds 18, 24, and 42 produced 58.9, 95.6 and 34 % inhibition of invasion, respectively. Note the logarithmic scale. **c** Real time PCR of the host cell GAPDH mRNA was used as a control for equal amounts present in each assay.

Twelve of the biochemically-validated compounds were assayed for their effects on parasite motility and infectivity. As shown in Fig. 5 and Additional file 6, two of the compounds, 24 and 42, had a pronounced effect on gliding motility when assayed at 500 μM against isolated *P. berghei* sporozoites.

These same compounds, as well as compound 18, impaired the ability of parasites to invade hepatocytes when tested at 500 μM in a sporozoite neutralization assay [41, 42]. As shown in Fig. 5 and Additional file 6, compounds 18, 24 and 42 produced 58, 95 and 34 % inhibition of hepatocyte invasion, respectively as assayed by RT-PCR of *P. berghei* 18S ribosomal copy number. Interestingly, eight of the compounds (1, 3, 5, 19, 21, 29, 32, and 36) produced a trend towards increased infectivity of sporozoites.

### Initial cytotoxicity study on human hepatocytes via flow cytometry

**Fig. 6:**
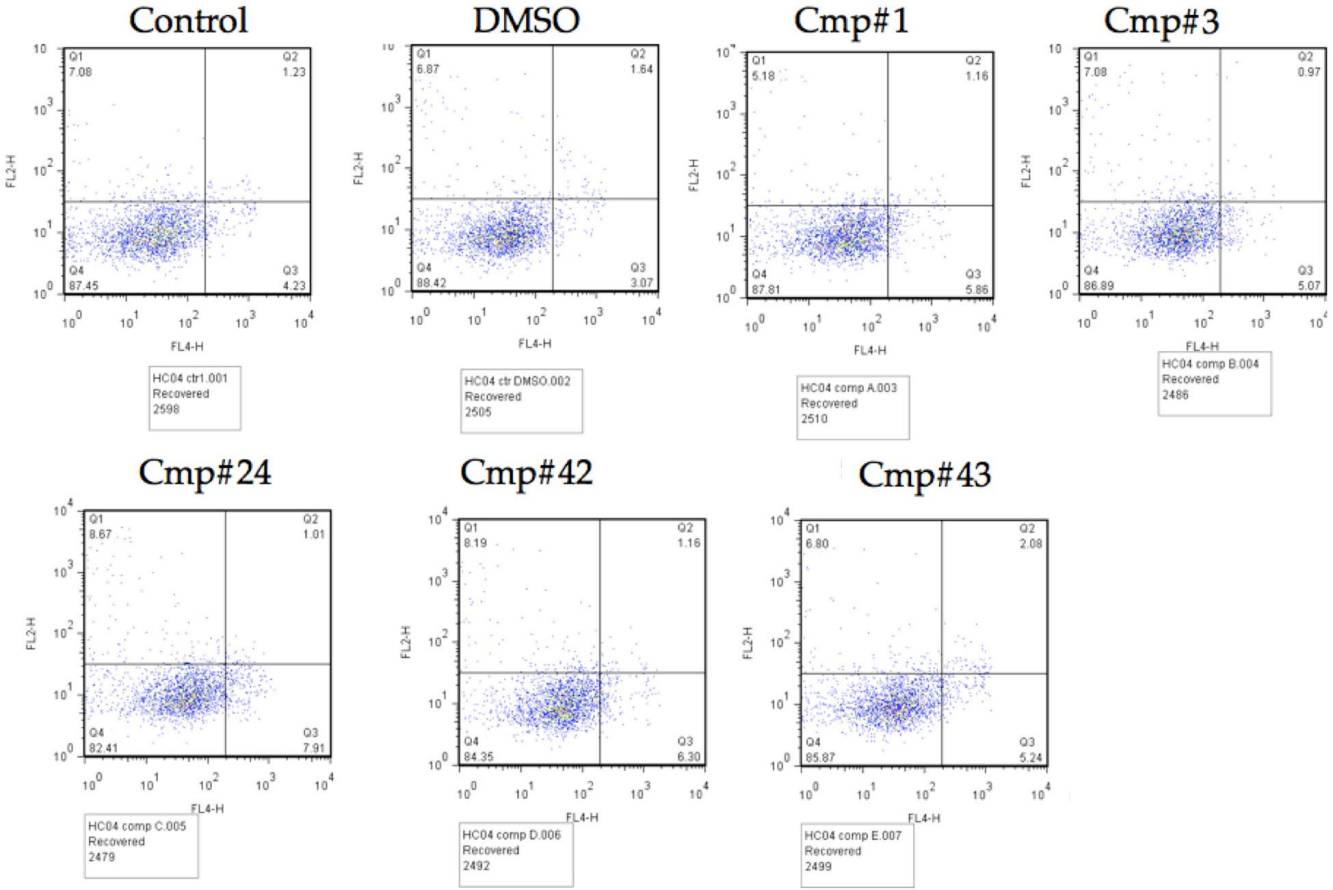
Evaluation of compound cytotoxicity on human hepatocytes. Human hepatocytes (HC-04 cells) were treated with 1 mM of the compounds, and then analysed for the expression of apoptotic markers [via Annexin V-APC (FL4) and Propidium Iodide (FL2) staining] by flow cytometry. Representative examples of a sub-set of the compounds tested. Recovered cells were then analysed for the expression of both apoptotic markers Annexin-V and PI. At this concentration, none of the compounds induced heightened expression of either apoptotic markers [via Annexin V-APC (FL4) and Propidium Iodide (FL2) staining] by flow cytometry. Note that for compound 24, the fluorescence peaks for Annexin-APC are shifted slightly to the right, indicating that at higher concentrations, some cytotoxicity would likely be observed. However, the compound dosage used here—1 mM—is already much higher than would typically be observed under physiological conditions.

Those compounds showing activity in vitro and in vitro parasite assays (Fig. 5; Additional file 6) were also assayed for their effects on the viability of human HC-04 liver cells. As shown in Fig. 6, preliminary studies using 1 mM of five selected compounds in 1 % final DMSO concentration do not show the induction of apoptotic markers as assayed by annexin V and phosphoinositol (PI)-staining after 96 h incubation. Taking these results together, they suggest that these compounds are indeed parasite-specific.

### Ternary co-crystal structure confirms mode of action of compound 24

**Fig. 7:**
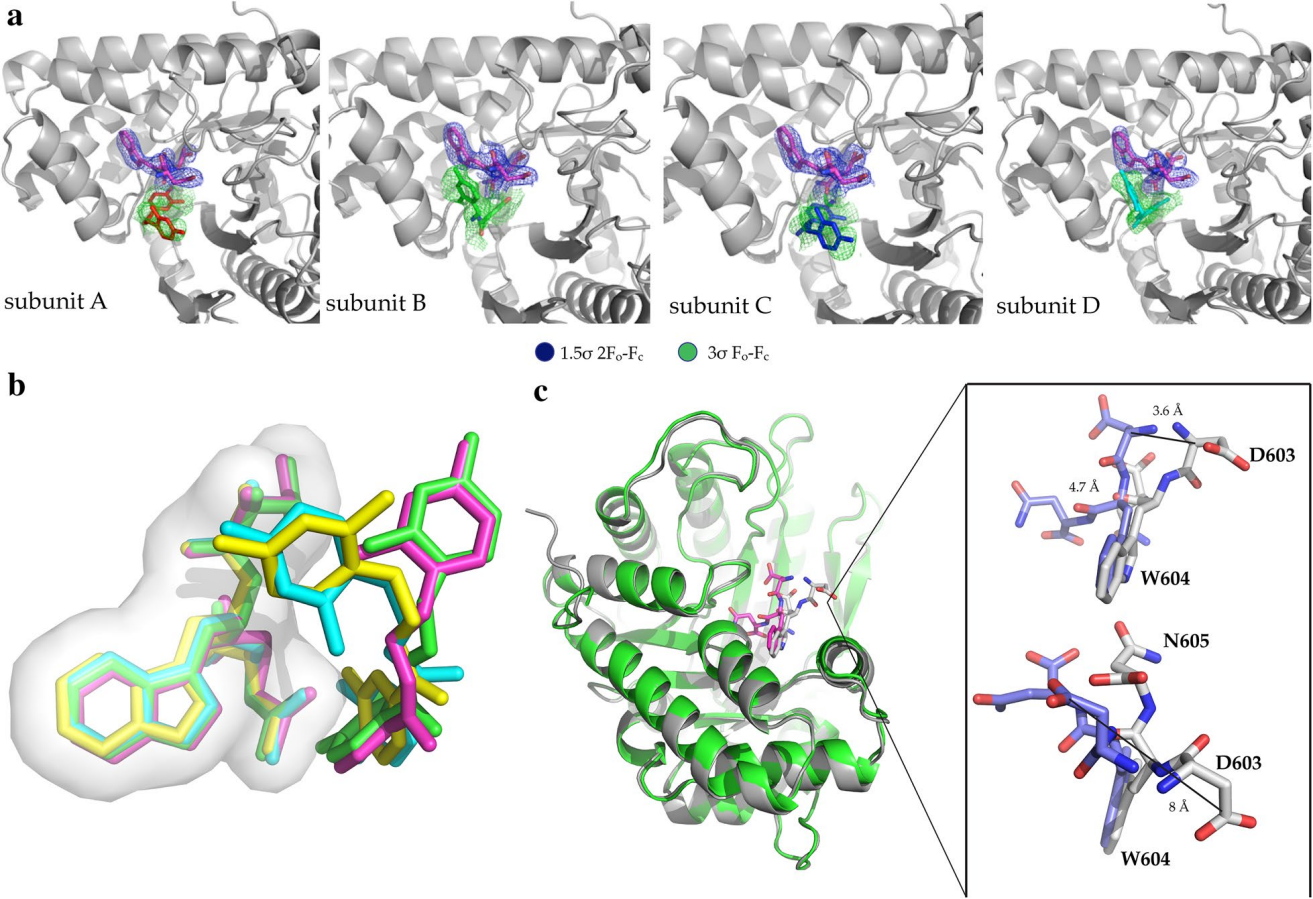
Ternary co-crystal structure of *Pf*Aldolase with TRAP and compound 24. **a** Panel showing all four sub-units of *Pf*Aldolase bound with TRAP (*blue* 2F_o_-F_c_ map at 1.5 s level) and compound 24 (*green* difference density map at 3 s level). **b** Superposition of compound 24 relative to the TRAP-tails, TRAP is represented as transparent surface. While all four TRAP-tails are in an almost identical position, compound 24 adopts two major conformations. **c** Structural comparison of the *Pf*Aldolase–TRAP complex 2PC4 (*grey* [26]) with *Pf*Aldolase–TRAP–Cmp 24 complex 4TR9 (*green* and *magenta*). Binding of compound 24 to the *Pf*Aldolase–TRAP interface results in a dramatic inward movement of TRAP compared to our previous structure of *Pf*Aldolase with TRAP alone (2PC4, [26]).

**Fig. 8:**
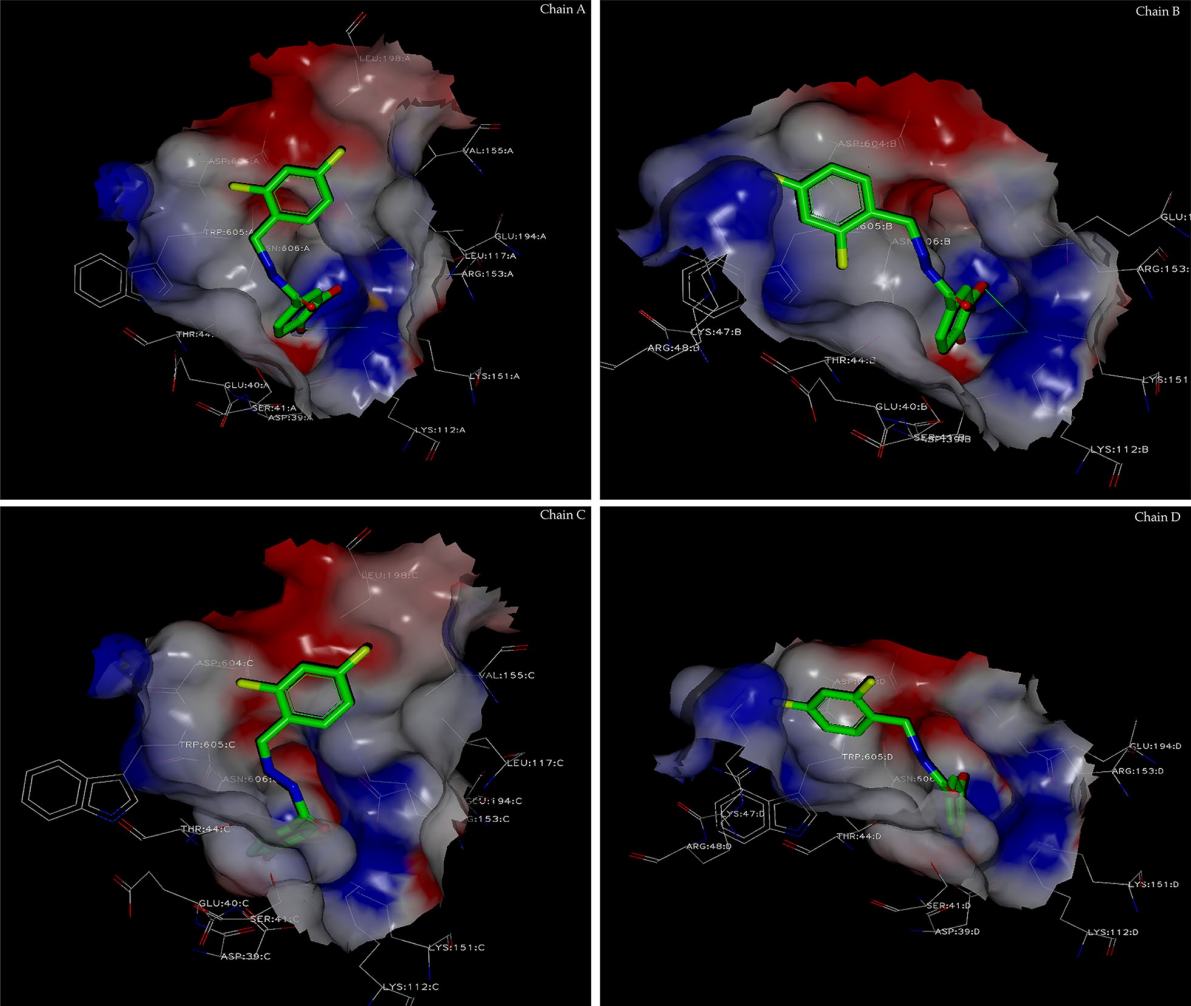
Detailed interaction representation of compound 24 with the *Pf*Aldolase–TRAP interface. The electrostatic surface of the mixed interface is shown and compound 24 is kept in a *green-stick* representation. Figure generated in Vida [71] and rendered with Povray [72].

Well-diffracting crystals of the ternary complex *Pf*Aldolase–TRAP were obtained in the presence of various compounds. However in only one case, that of compound 24, clear electron density was observed for both TRAP and the compound in all four sub-units of the aldolase tetramer (Fig. 7a; Additional files 7, 8, 9, 10). Notably, in the previously published aldolase–TRAP co-crystal structure (PDB ID: 2pc4) [26], TRAP could only be seen in one out of four aldolase sub-units, further highlighting the stabilizing effect of this compound on the complex. It is worth noting that the superposition comparison between 2pc4 [26] and the aldolase–TRAP–compound 24 ternary complex reveals significant shifts of the TRAP-binding position upon addition of compound 24. The W604 ring system varies only slightly in its position by 0.8 Å (Fig. 7b), whereas the D603 C_a_-position shifts by 3.6 Å and the N605 C_a_-position by 4.7 Å (Fig. 7c). The largest observed distance between the two crystal structures is the side chain of residue D603 with 8 Å. Overall, the TRAP-tails are pushed further into the pocket of aldolase upon addition of the small molecule stabilizer, compound 24, thereby occluding the F16P substrate-binding site (Additional file 11). The dihydroxybenzyl-ring of compound 24 stays within the experimental error of the X-ray crystal structure in the same position in all four sub-units, contacting residue N606 of TRAP and N39, E40, T44 of aldolase through hydrophobic interactions. The dichlorobenzyl-ring system adopts two major conformations, indicating flexibility of the compound when bound to the *Pf*Aldolase–TRAP interface (Figs. 7c, 8, Additional files 3, 4, 5, 6, 7, 8, 9, 10). The contacting residues on aldolase mainly provide hydrophobic interactions mediated through residues L117, R158 and L198 for binding mode 1 and T44, K47 and R48 in the alternative conformation, while also contacting D604 of the TRAP-tail. A future derivative of compound 24 with a double ring system at this position may confer higher binding affinities to the *Pf*Aldolase–TRAP complex by decreasing the rotational freedom of the compound in the binding site.

When comparing the predicted binding pose of compound 24 from VLS with the actual experimentally observed in the crystal structure, one can observe a reasonable good agreement of the proximity of the ligand (Additional file 12). The motion of the TRAP-peptide deeper into the *Pf*Aldolase active site introduces significant changes that currently cannot be computationally predicted a priori. When re-docking compound 24 to the actual observed ternary co-crystal structure (4TR9), the predicted pose results in a better overlap, with a preference for the binding mode 2 where the dichlorobenzyl-ring system is in contact with T44, K47 and R48 (Additional file 12).

### Compound 24 does not cross-react with rabbit aldolase

**Fig. 9:**
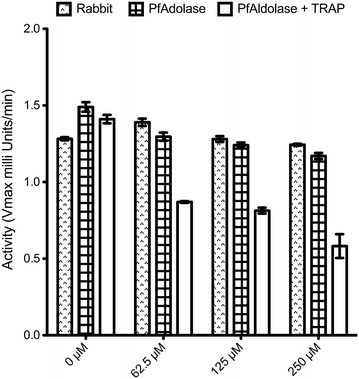
Inhibition assay of Rabbit and *Plasmodium* aldolase with compound 24. Rabbit aldolase was utilized as a surrogate for human aldolase due to the sequence identity. Different concentrations of compound 24 were tested in the presence and absence of the TRAP-peptide. No apparent inhibition is observed in either rabbit or *Plasmodium* aldolase alone, while a strong inhibition is observed when TRAP is present. Assays were performed in triplicate under standard conditions.

To validate that this initial hit compound represents a viable drug candidate, enzymatic assays were performed with rabbit aldolase, which is 99 % sequence identical to human aldolase (Additional file 1). Only four residues out of 364 vary between these two species, while *Plasmodium* shares only 56 % sequence identity (203/364 identical residues) with rabbit aldolase or human aldolase (Additional file 1). A dilution series of compound 24, ranging from 250 to 0 µM was tested with rabbit aldolase, *Pf*Aldolase, and *Pf*Aldolase in the presence of the TRAP-peptide. While compound 24 did not induce a significant change in activity in the rabbit aldolase or *Pf*Aldolase alone, a dramatic change in activity was observed upon addition of both the TRAP-peptide and compound 24 to *Pf*Aldolase, further supporting the initial hypothesis of compound 24 acting as a stabilizing agent of the *Pf*Aldolase–TRAP protein–protein interaction (Fig. 9). Compound 24 inhibits *Pf*Aldolase catalytic activity only in the presence of TRAP.

### Surface plasmon resonance (SPR) studies indicate decreased dissociation rates of the *Pf*Aldolase–TRAP complex in the presence of compound 24

**Fig. 10:**
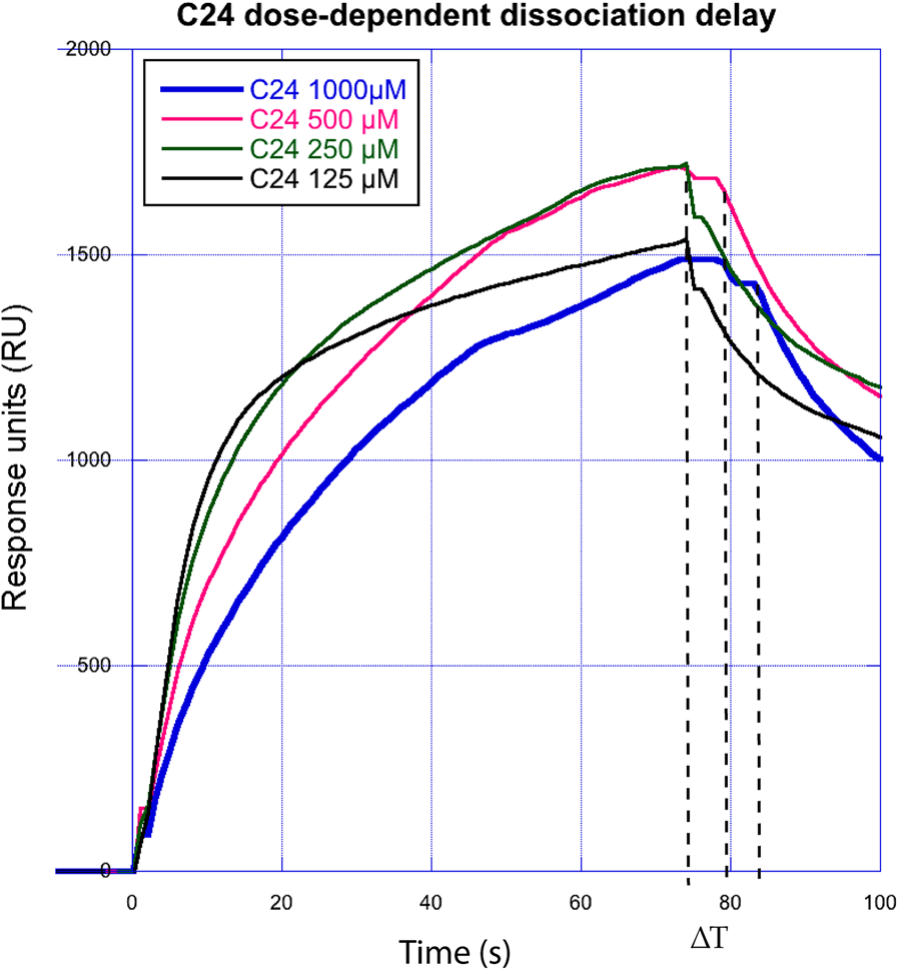
Delayed dissociation of TRAP bound to *Pf*Aldolase measured via SPR. Dose-dependent dissociation delay of PfTRAP-tail from *Pf*Aldolase immobilized on an SPR chip. A fixed amount of *Pf*TRAP-tail fused via a linker to Maltose binding protein was measured in the presence of increasing concentrations of compound 24 using SPR. Indicated with *dashed lines* is the dissociation delay observed at concentrations greater than 500 µM of compound 24.

The authors recently devised a method [34] by which screening and characterization of small molecules that enhance the binding of *Pf*Aldolase and TRAP can be performed. The binding of either *Pf*Aldolase to immobilized TRAP peptides or binding of TRAP to immobilized *Pf*Aldolase on a SPR chip has been previously demonstrated [34, 45]. In this study *Pf*Aldolase was passed over a chip with immobilized TRAP-peptides to measure the binding and dissociation in the presence of compound 24 (Fig. 10). The association constant (K_a_) decreases and the dissociation rate (K_d_) increases in the presence of compound 24, thereby indicating a stabilizing effect on the *Pf*Aldolase–TRAP complex under physiological buffer conditions. A dose-dependent delay in dissociation of ~5 s can be observed with concentrations >500 µM as indicated by dotted lines in Fig. 10. At the highest concentration tested of 1,000 µM the dissociation is delayed by ~10 s, providing a biophysical real time observation of the binding promoting capabilities of compound 24. No delayed dissociation effect is observed with lower concentrations of the compound than those depicted.

## Discussion

**Fig. 11:**
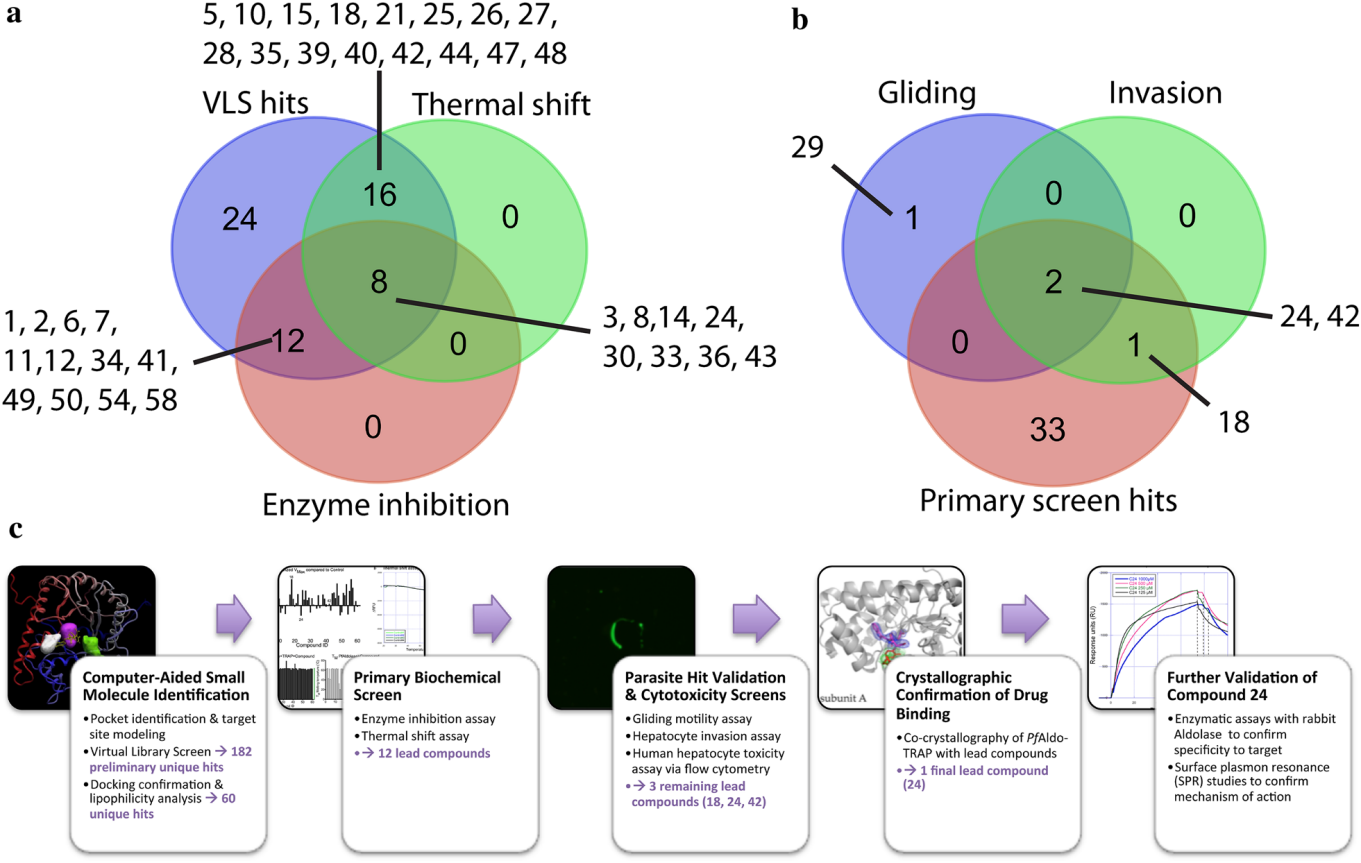
Triaging of VLS hits via primary screen and validation through secondary parasite assays. **a** Venn diagrams of our primary screen using VLS, enzyme activity assay and thermal shift data. 36 compounds showed activity in more than one assay. **b** Venn diagram of those compounds overlapping in at least two assays in our primary screens, gliding motility assay and liver invasion assay. Compound 24 emerges from five different assays and was the only one successfully visualized through our co-crystallization efforts. **c** Flowchart depicting the sequence of steps in the lead identification and verification process described here. While initially 315,105 chemical structures were screened using VLS, after each step fewer viable drug candidates remained. At the end of this sequence of computational and biochemical experiments, compound 24 emerged as the most promising lead.

Here, the challenge to pharmacologic targeting of a site strongly conserved in amino acid sequence between human and parasite in the key aldolase protein of the *Plasmodium* glideosome was overcome by successfully designing a screen for compounds that specifically stabilize a key protein–protein interface between *Pf*Aldolase and TRAP in the malaria parasite. The proposed binding mode of the stabilizing compound was confirmed in the ternary Aldolase–TRAP–C24 co-crystal structure (Figs. 7, 8, Additional files 7, 8, 9). Since the compounds also inhibited parasite motility and invasion (Figs. 5, 11, Additional file 6), they suggest that this interface in the glideosome is a relevant target for anti-malarial drug design. Furthermore, an additional effect of stabilizing the *Pf*Aldolase–TRAP interface may result in the occlusion of the actin-binding site to *Pf*Aldolase and thereby preventing actin binding, which is needed for a productive invasion event, hence potentiating the effect of the stabilizing ligand. Importantly, by stabilizing TRAP bound to aldolase, compound 24 also potentiates aldolase inhibition by the TRAP protein (Figs. 7, 8, Additional file 9), enhancing their appeal as drug candidates. A ternary complex of aldolase–TRAP and compound 24 can be rendered catalytically inactive with appropriate compound concentrations, thereby having a limiting effect on energy production of the parasite through glycolysis. Further structural and biochemical studies should help delineate the extent to which inhibition of glycolysis contributes to the compounds’ anti-infective effect versus direct interference with the glideosome’s physical mechanism of movement generation.

Of note, recent studies have called into question the conservation of the gliding machinery across *Apicomplexa*, and in particular the role played by aldolase in infectivity. Shen et al. showed that one aldolase isozyme (*Tg*ALD1) can be knocked out, and the altered parasites retain infectivity, although to a much lower degree [46]. In contrast, a recent study utilizing biolayer interferometry, as well as co-sedimentation studies, confirmed that aldolase and actin are required for *Plasmodium* for cell invasion of host cells [47], although this individual study is not considered definitive. The reader is referred to a recent comprehensive review discussing the similarities and differences of the glideosomes in *Toxoplasma* and *Plasmodium* [48] In addition to representing potential new leads in anti-malarial drug design, the compounds and methods described here represent an important new strategy for the field by providing new pharmacology (non-genetic) tools that may help clarify the *Toxoplasma* genetic studies. The most selective compounds could be valuable non-genetic tools for further investigating glideosome function in *Plasmodium*. At the concentrations tested, compound 24 did not inhibit rabbit aldolase, which was utilized as a surrogate for human aldolase with 99.3 % sequence identity (Fig. 9; Additional file 1). In addition, our studies suggest that the genetic findings in *Toxoplasma* may not apply to other biomedically important members of the *Apicomplexan* phylum [26].

Although proof-of-principle was achieved, the compounds exhibit relatively low potencies in functional assays, which is a limitation for their development into drug leads. Nevertheless, the potency of the hits themselves may be less important than validation of the target drug-binding pocket by structure-based screening: the pharmacophore space that is now mapped out by the hits and their bioactivities, in conjunction with the known interactions within the X-ray crystal structure, can serve as a blueprint for rational optimization of the hits in multiple directions (better potency, better bioavailability, etc.), using resources such as medicinal chemistry and fragment-based tethering. Notably, the GSK TCAMS [49] Novartis-GNF Malaria Box, and St Jude Children’s Research Hospital [50] datasets of hits from whole-cell screenings against *P. falciparum* blood stages include several compounds containing the *N*-(benzylideneamino)benzamide scaffold between them (Additional file 13). This scaffold is shared by some of the hits identified here (Fig. 4a), and medicinal chemistry derivatization of this scaffold may yield additional compounds with greater potency against the parasite or other favourable drug properties. It should also be noted that the average concentration of FDA approved drugs used to treat malarial disease in humans is close to 500 µM. For example, when injected intravenously the usual formulation of Chloroquine employed is 200 mg/ml, resulting in approximately 100 µM final concentration in the blood. Malarone, a combination therapy of atovaquone and proguanil is given as an oral dose at 750 mg/5 ml (~410 mM), corresponding to approximately 30 µM final concentration for a 60-kg person, assuming equal biodistribution throughout the body [51].

One intuitive, theoretical concern related to this study is that compounds targeted to the *Pf*Aldolase–TRAP interface may actually destabilize the complex or that stabilizing the target interface might enhance motility and infectivity. Indeed, several compounds showed destabilization on thermal shift. Notably, only the last three residues of TRAP (604–605) were visible in the crystal structure and receptor models which were screened against here. It is possible that these compounds interact in an inhibitory way with upstream TRAP residues that could not be accounted for in this screen. If so, these compounds may be the basis for a new approach to inhibit the glideosome as they should be even more specific. Destabilizing compounds, although not desired by the present design, may actually be useful from a drug development point of view once their exact mechanism of action is known. Additionally, eight compounds showed insignificant trends towards increases in invasion despite no change or decreases in motility, possibly indicating that some of the compounds may have additional off-target effects or change the *Pf*Aldolase–TRAP interaction in a way that increases invasion but not motility. As the precise chemistry and mechanism of the glideosome is still obscure, these possibilities cannot be ruled out. Compounds having this effect may nevertheless be useful non-genetic tools for studying precise glideosome sub-mechanisms.

Most drugs in use today inhibit biological interactions. However, the scientific literature contains several examples of biologically active small molecules that function by stabilizing protein–protein interactions in a bipartite manner, including fungal toxins [52–54], chemotherapeutic agents [55–59], antibiotics [60, 61], and immunosuppressants [62]. These examples indicate, and the results here ultimately suggest, that it could be possible to develop clinically useful compounds that enhance PPI. For a small molecule to inhibit a PPI, it must bind to its receptor with a higher affinity than, and at least similar specificity to, the protein’s native ligand. A vast collection of failed drug candidates demonstrates how difficult it is to compete with eons of evolutionary pressure that produced the biomolecular interaction in the first place [63]. Stabilizing that interaction, however, does not require competing with nature, as a nearby region is targeted by the small molecule. Rather, this approach tries to nudge the interaction’s equilibrium in the direction that is thermodynamically favoured to begin with. Thus a candidate enhancer does not need to bind either member of a protein complex with particularly high affinity—it is the aggregate of affinities of the proteins for each other and for the drug that matter [54]. As demonstrated by the compounds discovered here, adding just one or two contact points to a protein complex can make a very big difference in its stability.

The enhancer approach may work especially well for situations in which the conformational dynamics of a protein complex are key to its function. In this case, the ability of the glideosome to provide the motive force is dependent on the highly coordinated interactions of its members. Aldolase must tightly bind both actin and TRAP to allow motion to begin, but it must also rapidly release the TRAP tail after its cleavage to allow motion to continue. While it is unclear if the same pool of aldolase participates in both motility and glycolysis, the enzymatic binding and cleavage of F16P is crucial for providing the ATP molecules necessary for the actin-myosin power stroke [29, 64]. The various conformations of aldolase, TRAP, actin, and MyosinA must therefore exist in the ideal equilibriums to promote the proper bind-and-release sequences for each of the glideosome interactions. Shifting these equilibrium in either direction by inhibiting or enhancing any of the interactions involved should affect the motor. The computer-aided, structure-based approach to drug discovery presented here allowed the specific targeting of structural differences between multiple conformations of aldolase in order to shift the apo-aldolase/aldolase-F16P/aldolase–TRAP equilibrium towards the aldolase–TRAP complex in a parasite-selective fashion. Nature abounds with similar vulnerable systems of exquisitely regulated biological motors and complexes, many of which might be targeted by this structure-based enhancer method.

Future modelling and crystallographic studies should help define additional receptor pockets and conformations that can be exploited to design compounds that target different aspects of the glideosome, including the glideosome homologs present in different stages of the *Plasmodium* life-cycle, as well as glideosome components conserved in other apicomplexan pathogens, such as *Toxoplasma gondii* and *Cryptosporidium* spp. For example, given sufficient structural information, the interactions between MTIP and Myosin A in *Plasmodium* could also be targeted in a similar fashion to the TRAP–aldolase complex by either stabilizing the close conformation or preventing opening of the EF-hands of MTIP. Several structures of *Pf*MTIP and *Pk*MTIP in complex with Myosin A [65–67] and stapled peptides [68, 69] have been described to date. Analysis of their structural flexibility may provide crucial insights towards targetable interfaces and pockets.

While stabilizing the *Pf*Aldolase–TRAP interaction may seem like an unusual approach for rational drug design, by targeting this joint surface, the avenue it opens to promoting parasite specificity—TRAP is not present in humans—may prevent the emergence of resistance. Additionally, resistance mutations in aldolase’s active site would be highly unfavourable as they would likely interfere with glycolytic energy generation. Starnes et al. [64] demonstrated in *Toxoplasma* that mutations near the active site of aldolase are not tolerated by the parasite, which is in agreement with the decreased likelihood of resistance mutations emerging.

The strategy of targeting a hybrid surface composed of a conserved target and a non-conserved target for the purposes of combating resistance may be broadly applicable to structure-based drug design. This may be especially useful for developing agents to fight eukaryotic pathogens, as many of their essential proteins have highly conserved human homologues, and are otherwise difficult to specifically target. It may also be possible to use this type of approach to design therapeutics for rapidly mutating viruses by selectively modulating host-pathogen interactions, i.e. preventing the dissociation of a viral surface protein with its host receptor may increase the virus’s vulnerability to other drugs or to the host’s own immune system. In this case, the druggable surface encompassing the viral protein would provide specificity, while the unlikelihood of mutations in the host protein may protect against the development of drug resistance. Similarly, one can envision targeting a complex of a normal housekeeping protein and a mutant oncoprotein to selectively kill cancer cells.

It is important to note that the compounds and chemical scaffolds identified here are not found among the anti-malarials currently in clinical use. The fact, that the identified compound emerged directly from a VLS effort and has not undergone any chemical optimization, while showing an on-target effect by various biophysical and parasitological assays bodes well for its future development from a probe to a lead compound. This effort, therefore, represents a successful ‘scaffold hop’ in anti-malarial drug discovery. If ultimately successful, these drugs and their derivatives would constitute a novel class or classes of anti-malarial agents, as well as the first drugs to target the aldolase–TRAP interaction.

At present, a more potent, safe drug with an identical mechanism of action to compound 24 could be useful for malaria prophylaxis, but its activity against merozoites, the extracellular form of the erythrocytic stage parasites was negative, likely due to the additional C-terminal extension of the merozoite TRAP homologue MTRAP occluding binding of compound 24. Nevertheless, if the compounds identified here do not cross-react with merozoites, a similar screen to that described in this study, targeting MTRAP, the merozoite homologue of TRAP, could yield a similar, specific drug active in the blood stages. Based on the sequence identity of the terminal residues of TRAP with CTRP, the circumsporozoite TRAP-like protein (PlasmoDB code PF3D7_0315200) expressed during the mosquito stage, it is anticipated that compound 24 may function as a stabilizer of the *Pf*Aldolase–CTRP interface and be able to block mosquito midgut invasion of the parasite. Such a compound could perhaps be used as a spray to treat bednets or to treat hatching areas and prevent spread of mosquitoes carrying the parasite.

## Conclusion

In summary, the results presented here validate the aldolase–TRAP interaction within the *Plasmodium* glideosome as a drug discovery target, by proving both that it can be pharmacologically targeted and that doing so does affect the parasite’s motility and invasion capabilities. It remains to be determined, if a similar approach can succeed in *Toxoplasma* and other *Apicomplexa*, but using the chemical probes discovered here may contribute to the understanding of the role of aldolase in *Toxoplasma* gliding motility.

This work also provides proof-of-concept that the structure-based, selective, enhancement of PPI is a viable, efficient and effective method of novel drug hit discovery, opening new avenues to drug discovery for challenging targets, such as the glideosome.

## Additional files

Additional file 1. Clustal-W sequence alignment of *Plasmodium*, human and rabbit aldolase. Additional file 2. Pockets in the unliganded aldolase crystal structure. Additional file 3. Receptor models for VLS and docking boxes. Additional file 4. VLS hit lists and compound structures and properties. Additional file 5. Stereo figures of selected VLS hit poses. Additional file 6. Sporozoite motility assay results for all compounds tested. Additional file 7. Ligplot representation of the TRAP peptides. Additional file 8. Ligplot representation of the Compound 24 molecules. Additional file 9. Stereo figures of each PfAldolase subunit bound to TRAP and compound 24. Additional file 10. Overview of the *Pf*Aldolase ternary complex structure. Individual *Pf*Aldolase subunits are colored by chain. The TRAP-peptide present in all four subunits is shown as stick representation as well as Compound 24. The movie zooms onto each sub-unit and represents the individual binding sites. At the end a sphere model of TRAP and Compound 24 is shown to illustrate possible areas, which could be filled during future SAR studies. Additional file 11. Superposition of *Plasmodium* aldolases apo, TRAP-bound and compound 24 and TRAP-bound form. Colour coding of the structure is kept identical to Fig. 5C. Additional file 12. Stereo figure of compound 24 in the crystal structure compared to the docking pose obtained with 2PC4. Additional file 13. Identified C24 scaffold homologs within the TCAMs dataset.
