# Supplementary material for: Inhibition by stabilization: targeting the Plasmodium falciparum aldolase–TRAP complex
Source: Malar J. 2015 Aug 20;14:324. doi: 10.1186/s12936-015-0834-9 (PMC4545932; doi:10.1186/s12936-015-0834-9)
Supplement: Additional file 1. — Clustal-W sequence alignment of Plasmodium, human and rabbit aldolase. [file 12936_2015_834_MOESM1_ESM.pdf]

Additional file 1: Clustal-W sequence alignment of *Plasmodium*, Human and Rabbit aldolase. Human and rabbit aldolase share 360/364 residues (99% sequence identity).

```
CLUSTAL W (1.83) multiple sequence alignment

HumanA          MPHSYPALS------AEQKKELSDIALRIVAPGKGILAADESVGSMAKRLSQIGVENTEENRRLYRQVLFSADDRVKKCI
RabbitC         MPHSYPALS------AEQKKELSDIALRIVAPGKGILAADESVGSMAKRLSQIGVENTEENRRLYRQVLFSADDRVKKCI
P.falciparum    MAHCTEYMNAPKKLPADVAEELATTAQKLVQAGKGILAADESTQTIKKRFDNIKLENTIENRASYRDLLFGTK-GLGKFI
                *.*.   :.      *:  :**:  * ::* .**********. :: **:.:* :*** ***  **::**.:.  : * *

HumanA          GGVIFFHETLYQKDDNGVPFVRTIQDKGIVVGIKVDKGVVPLAGTDGETTTQGLDGLSERCAQYKKDGADFAKWRCVLKI
RabbitC         GGVIFFHETLYQKDDNGVPFVHTIQDKGIVVGIKVDKGVVPLAGTDGETTTQGLDGLSERCAQYKKDGADFAKWRCVLKI
P.falciparum    SGAILFEETLFQKNEAGVPMVNLLHNENIIPGIKVDKGLVNIPCTDEEKSTQGLDGLAERCKEYYKAGARFAKWRTVLVI
                .*.*:*.***:**:: ***:*. ::::.*: *******:* :. ** *.:*******:*** :* * ** ***** ** *

HumanA          S--ERTPSALAILENANVLARYASICQQNGIVPIVEPEILPDGDHDLKRCQYVTEKVLAAVYKALSDHHVYLEGTLLKPN
RabbitC         S--ERTPSALAILENANVLARYASICQQNGIVPIVEPEILPDGDHDLKRCQYVTEKVLAAVYKALSDHHVYLEGTLLKPN
P.falciparum    DTAKGKPTDLSIHETAWGLARYASICQQNRLVPIVEPEILADGPHSIEVCAVVTQKVLSCVFKALQENGVLLEGALLKPN
                .  : .*: *:* *.*  *********** :*********.** *.:: *  **:***:.*:***.:: * ***:*****

HumanA          MVTPGHACPIKYTPEEIAMATVTALRRTVPPAVPGVTFLSGGQSEEEASFNLNAINRCPLPRPWALTFSYGRALQASALN
RabbitC         MVTPGHACPIKYSPEEIAMATVTALRRTVPPAVPGVTFLSGGQSEEEASLNLNAINRCPLPRPWALTFSYGRALQASALN
P.falciparum    MVTAGYECTAKTTTQDVGFLTVRTLRRTVPPALPGVVFLSGGQSEEEASVNLNSINAL-GPHPWALTFSYGRALQASVLN
                ***.*: *. * :.:::.: ** :********:***.************.***:**    *:***************.**

HumanA          AWRGQRDNAGAATEEFIKRAEVNGLAAQGKYEGSGEDGGAAAQSLYIANHAYL
RabbitC         AWRGQQDNAGAATEEFIKRAEVNGLAAQGKYEGSGEDGGAAAQSLYIANHAY-
P.falciparum    TWQGKKENVAKAREVLLQRAEANSLATYGKYKG-GAGGENAGASLYEKKYVY-
                :*:*:::*.. * * :::***.*.**: ***:* * .*  *. ***  ::.*
```
