# Supplementary material for: Inhibition by stabilization: targeting the Plasmodium falciparum aldolase–TRAP complex
Source: Malar J. 2015 Aug 20;14:324. doi: 10.1186/s12936-015-0834-9 (PMC4545932; doi:10.1186/s12936-015-0834-9)
Supplement: Additional file 2. — Pockets in the unliganded aldolase crystal structure. [file 12936_2015_834_MOESM2_ESM.pdf]

Additional file 2: Pockets in the unliganded aldolase crystal structure (PDB ID: 1a5c). Pocket numbers correspond to those in Figure 1C of the main text. Key pockets are highlighted, and non-conserved residues contacting each pocket are listed in bold.

Pocket #	Volume (Å^3)	Area (Å^2)	Radius (Å)	Nonsphericity	Aldolase Residues Contacting Pocket	TRAP Residues Contacting Pocket
1	239.11	325.36	3.85	1.75	Q26, V29-G32, E231, P269, A270, P272, N294, A295, G297-H299, W301-T304, T344, Y345, K347	
2	281.97	326.56	4.07	1.57	E196-A199, C208, N238-M239, T241-Y244, C246-A248, T250-T252, V255, L277-Q281, E285, N289	
3	227.86	255.08	3.79	1.41	S41, T44, F84, **E85**, K112-**L117**, R138, Y142, R153	**N606**
4	109.52	179.08	2.97	1.62	K27, Q30, K33, H103, N106-P109, R148	
5	133.22	169.82	3.17	1.35	P10-L13, R179, S182, I183, Q185, Q186, N226-V228	
6	188.41	174.08	3.56	1.10	A37, D39, E40, K151, E194, K236, L277-G279, S306-A310	**W605, N606**
7	118.41	156.06	3.05	1.34	N60, S63, D66, N92, E93, W319, G321, K323, V326	
8	102.94	126.75	2.91	1.19	K47, R48, **N51**, I52, R309, A313, L316, N317	**W605**
