# Supplementary material for: Inhibition by stabilization: targeting the Plasmodium falciparum aldolase–TRAP complex
Source: Malar J. 2015 Aug 20;14:324. doi: 10.1186/s12936-015-0834-9 (PMC4545932; doi:10.1186/s12936-015-0834-9)

Additional file 3: Receptor models for VLS and docking boxes.

Aldolase is shown as a ribbon colored in a gradient from blue at its N-terminus to red at its C-terminus. TRAP peptides are shown in ball-and-stick depiction with standard atom-coded colors (oxygen = red, nitrogen = blue, hydrogen = grey, carbon = yellow). The red balls represent the initial docking “probe,” which is where each ligand was placed at the start of each docking procedure. The VLS “boxes” in purple represent the regions in which the docking algorithm concentrated its search. (a) The “2pc4 model” receptor. The green graphical object depicts the pocket around which the VLS boxes were initially built. (b) The “falciparum model” receptor. (c) The “gapped-pocket model” receptor.

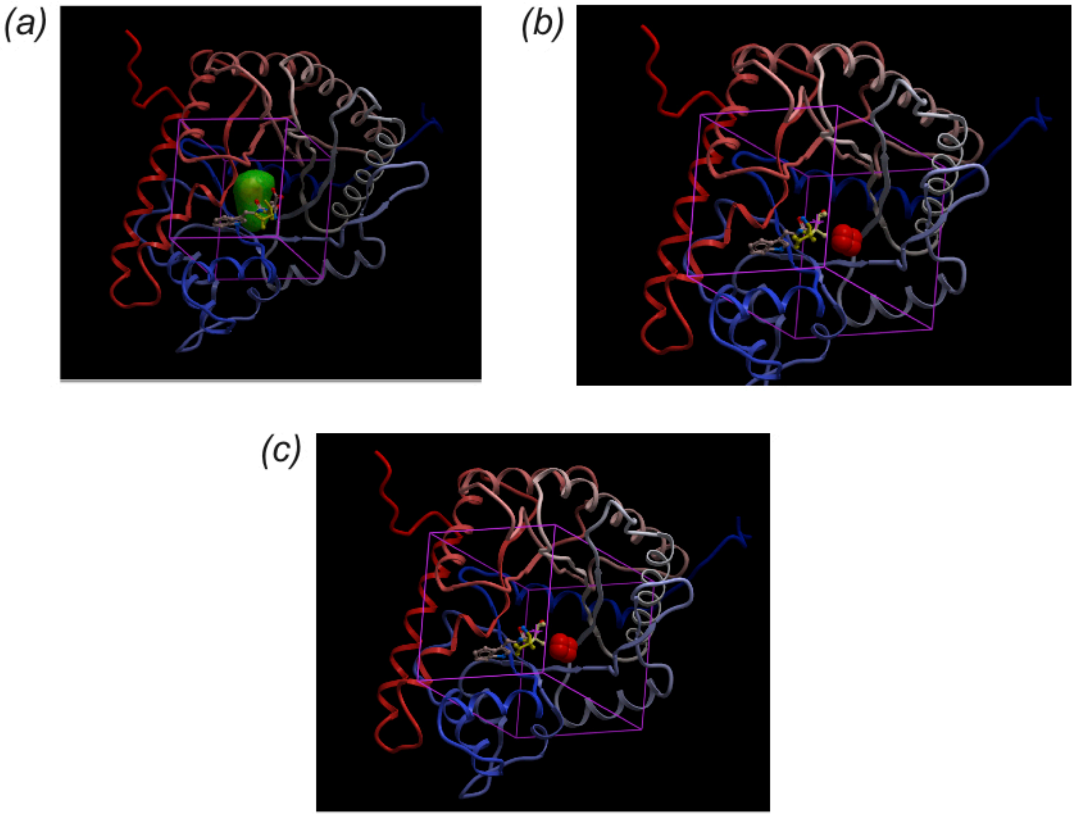

Supplement: Additional file 3. — Receptor models for VLS and docking boxes. [file 12936_2015_834_MOESM3_ESM.pdf]
