# Supplementary material for: Inhibition by stabilization: targeting the Plasmodium falciparum aldolase–TRAP complex
Source: Malar J. 2015 Aug 20;14:324. doi: 10.1186/s12936-015-0834-9 (PMC4545932; doi:10.1186/s12936-015-0834-9)
Supplement: Additional file 4. — VLS hit lists and compound structures and properties. [file 12936_2015_834_MOESM4_ESM.pdf]

Additional file 4:

Supplemental Data - VLS hitlists and Compound Structures and Properties

>300,000 compounds were screened against 3 models of the *Plasmodium* aldolase-TRAP complex. The following tables list the final sets of compounds obtained for each receptor:

The table headings are defined as follows:

- Compound #: a unique number (1-60) assigned to each compound
- VLS score: the lowest energy score obtained for each compound after 3 rounds of screening against the receptor model
- Docking score: the energy score obtained for each compound when it was re-docking to its receptor using a slower, more robust docking algorithm
- Score difference: (Docking score) – (VLS score)
- RMSD: root-mean-squared deviation of the docked position and conformation of the compound between the VLS and re-docking runs

VLS hits for "2pc4 model"

Compound #	VLS Score	Docking Score	Score Difference	RMSD (Å)
4	-37.50	-36.92	0.58	0.08
40	-34.07	-37.10	-3.03	0.00
44	-43.32	-43.28	0.05	0.00
45	-46.36	-36.59	9.77	1.98
47	-36.64	-37.11	-0.47	2.14
56	-32.92	-32.18	0.75	1.73

VLS hits for "falciparum model"

Compound #	VLS Score	Docking Score	Score Difference	RMSD (Å)
3	-36.51	-36.50	0.01	0.00
5	-33.66	-33.42	0.24	0.75
15	-36.22	-33.04	3.18	0.21
22	-33.63	-35.83	-2.20	0.05
32	-37.62	-34.41	3.21	0.48
37	-34.12	-33.16	0.96	0.09
40	-33.30	-36.58	-3.28	0.08
43	-32.39	-32.34	0.06	0.02
46	-32.55	-32.56	-0.01	0.00
53	-32.21	-32.23	-0.03	0.01
55	-37.23	-37.01	0.22	0.10

VLS hits for “gapped-pocket model”

Compound #	VLS Score	Docking Score	Score Difference	RMSD (Å)
1	-32.43	-33.19	-0.76	0.03
2	-36.41	-42.87	-6.47	0.07
5	-36.46	-36.53	-0.06	0.75
6	-37.08	-36.52	0.56	0.03
7	-35.10	-35.59	-0.49	0.05
8	-33.05	-33.11	-0.06	0.03
9	-36.10	-33.41	2.69	0.17
10	-35.37	-35.32	0.05	1.87
11	-32.71	-32.41	0.30	1.70
12	-35.11	-32.30	2.81	2.72
13	-33.02	-34.97	-1.95	2.12
14	-35.00	-34.46	0.54	0.01
16	-35.12	-34.91	0.21	0.00
17	-34.25	-34.32	-0.07	0.02
18	-35.05	-34.92	0.13	0.00
19	-34.49	-34.12	0.37	0.00
20	-34.78	-32.09	2.69	2.22
21	-35.30	-35.28	0.02	0.03
23	-37.96	-43.33	-5.37	0.09
24	-32.67	-32.79	-0.11	0.03
25	-32.61	-32.44	0.17	0.01
26	-37.45	-37.65	-0.20	0.00
27	-35.54	-41.36	-5.83	0.05
28	-34.21	-33.17	1.03	2.21
29	-35.18	-42.15	-6.97	2.27
30	-33.65	-33.36	0.30	1.81
31	-34.27	-33.76	0.51	0.02
33	-39.25	-38.68	0.57	0.03
34	-36.53	-36.82	-0.29	0.00
35	-32.47	-33.42	-0.95	1.81
36	-33.16	-32.87	0.29	0.01
38	-35.19	-35.18	0.01	0.00
39	-38.55	-37.22	1.34	0.04
41	-38.86	-38.45	0.41	0.00
42	-34.08	-34.01	0.08	0.00
48	-32.34	-33.15	-0.81	4.14
49	-37.85	-37.39	0.46	0.03
50	-32.98	-36.85	-3.87	0.68
51	-37.08	-37.01	0.08	0.03
52	-38.58	-37.99	0.60	6.84
54	-35.14	-41.21	-6.06	0.09
57	-35.55	-35.89	-0.34	0.00
58	-33.41	-33.16	0.25	1.66

Compound #	VLS Score	Docking Score	Score Difference	RMSD (Å)
59	-32.74	-32.69	0.05	2.26
60	-34.63	-32.09	2.53	0.02

Compound Structures & Properties

The compounds in the ChemBridge (San Diego, CA) screening library are generally uncharacterized. However, several physical and chemical features including solubility, hydrophobicity, flexibility, polarity, etc., can be predicted based on the compounds' formula and molecular structure. These features are listed below for each of the final 60 VLS hits.

Compound # 1

Chembridge ID 5112957

MW 270.3
Formula C15 H14 N2 O3
Name 4-hydroxy-N'-(4-methoxybenzylidene) benzohydrazide

LogSW -2.453
RB 3
tPSA 70.92
hDon 2
hAcc 4
cLogP 2.18

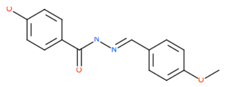

molLogP 3.01264

SMILES C(C=C(O)C=C1)=C1C(=O)N/N=C/C(C=CC(=C1)OC)=C1

Compound # 2

Chembridge ID 5112979

MW 285.3
Formula C14 H11 N3 O4
Name 4-hydroxy-N'-(2-nitrobenzylidene) benzohydrazide

LogSW -2.799
RB 3
tPSA 104.83
hDon 2
hAcc 5
cLogP 2

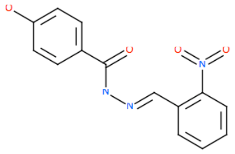

molLogP 2.47206

SMILES O=N(=O)C(C=CC=C1)=C1/C=N/NC(=O)C(C=CC(O)=C1)=C1

Compound # 3

Chembridge ID 5121901

MW 192.2
Formula C9 H12 N4 O
Name 1-(4-aminophenyl)-1-ethanone semicarbazone

LogSW -1.833
RB 2
tPSA 93.5
hDon 3
hAcc 2
cLogP 1.44

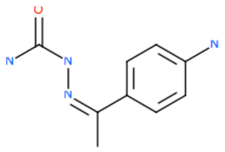

molLogP .889302

SMILES C(C=C(N)C=C1)=C1/C(=N\NC(=O)N)C

Compound # 4

Chembridge ID 5153389

MW 223.2
Formula C9 H9 N3 O4
Name N'-acetyl-2-nitrobenzohydrazide

LogSW -2.179
RB 2
tPSA 101.34
hDon 2
hAcc 4
cLogP -.41

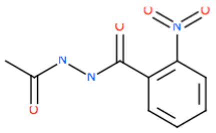

molLogP .041457

SMILES O=N(=O)C(C=CC=C1)=C1C(=O)NNC(=O)C

MW: molecular weight, g/mol	hDon: # of hydrogen-bond donors
LogSW: predicted aqueous solubility	hAcc: # of hydrogen-bond acceptors
RB: # of rotatable bonds	cLogP: predicted octanol-water partition coefficient (Chembridge algorithm)
tPSA: total polar surface area (Angstroms^2)	molLogP: predicted octanol-water partition coefficient (Molsoft algorithm)
	SMILES: Simplified Molecular Input Line Entry Specification

Compound # 5

Chembridge ID 5153799

MW 225.2

Formula C9 H7 N O4 S

Name 8-hydroxy-5-quinolinesulfonic acid

LogSW .343

RB 1

tPSA 87.49

hDon 2

hAcc 5

cLogP -.71

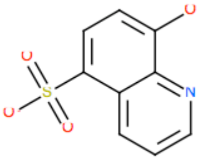

molLogP .616233

SMILES C(C1)=C(C2C(C=1O)=NC=CC=2)S(=O)(=O)[O-]

Compound # 6

Chembridge ID 5227275

MW 325.2

Formula C12 H9 Br N2 O2 S

Name N'-[(5-bromo-2-thienyl)methylene]-4-hydroxybenzohydrazide

LogSW -3.391

RB 3

tPSA 61.69

hDon 2

hAcc 3

cLogP 2.81

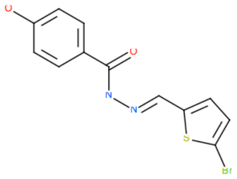

molLogP 3.62692

SMILES C(C=C(S1)/C=N/NC(=O)C(C=CC(=C2)O)=C2)=C1[Br]

Compound # 7

Chembridge ID 5227278

MW 291.3

Formula C12 H9 N3 O4 S

Name 4-hydroxy-N'-[(5-nitro-2-thienyl)methylene]benzohydrazide

LogSW -2.62

RB 3

tPSA 104.83

hDon 2

hAcc 5

cLogP 1.74

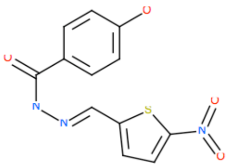

molLogP 2.75402

SMILES C(C=C(S1)/C=N/NC(=O)C(C=CC(=C2)O)=C2)=C1N(=O)=O

Compound # 8

Chembridge ID 5227280

MW 260.3

Formula C13 H12 N2 O2 S

Name 4-hydroxy-N'-[(3-methyl-2-thienyl)methylene]benzohydrazide

LogSW -2.569

RB 3

tPSA 61.69

hDon 2

hAcc 3

cLogP 2.4

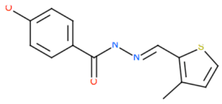

molLogP 3.07727

SMILES C(=N/NC(=O)C(C=CC(O)=C1)=C1)/C(SC=C1)=C1C

MW: molecular weight, g/mol	hDon: # of hydrogen-bond donors
LogSW: predicted aqueous solubility	hAcc: # of hydrogen-bond acceptors
RB: # of rotatable bonds	cLogP: predicted octanol-water partition coefficient (Chembridge algorithm)
tPSA: total polar surface area (Angstroms^2)	molLogP: predicted octanol-water partition coefficient (Molsoft algorithm)
	SMILES: Simplified Molecular Input Line Entry Specification

Compound # 9

Chembridge ID 5227289

MW 286.3
Formula C15 H14 N2 O4
Name 4-hydroxy-N'-(2-hydroxy-3-methoxybenzylidene)benzohydrazide

LogSW -1.938
RB 3
tPSA 91.15
hDon 3
hAcc 5
cLogP 1.44

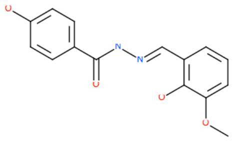

molLogP 2.51318

SMILES C(=N/NC(=O)C(C=CC(O)=C1)=C1)/C(C=CC1)=C(C=1OC)O

Compound # 10

Chembridge ID 5227292

MW 229.2
Formula C12 H11 N3 O2
Name 4-hydroxy-N'-(1H-pyrrol-2-ylmethylene)benzohydrazide

LogSW -1.027
RB 3
tPSA 77.48
hDon 3
hAcc 3
cLogP .86

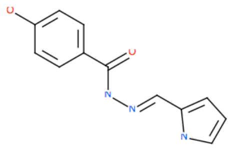

molLogP 1.87264

SMILES N(C=CC1)C=1/C=N/NC(=O)C(C=CC(=C1)O)=C1

Compound # 11

Chembridge ID 5227298

MW 286.4
Formula C15 H14 N2 O2 S
Name 4-hydroxy-N'-[4-(methylthio)benzylidene]benzohydrazide

LogSW -3.117
RB 3
tPSA 61.69
hDon 2
hAcc 3
cLogP 2.82

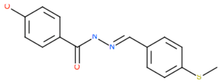

molLogP 3.60632

SMILES C(C=C(O)C=C1)=C1C(=O)N/N=C/C(C=CC(=C1)SC)=C1

Compound # 12

Chembridge ID 5227303

MW 256.3
Formula C14 H12 N2 O3
Name 4-hydroxy-N'-(3-hydroxybenzylidene)benzohydrazide

LogSW -1.847
RB 3
tPSA 81.92
hDon 3
hAcc 4
cLogP 1.59

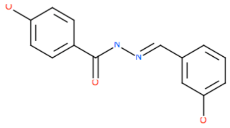

molLogP 2.66317

SMILES C(C=C(O)C=C1)=C1C(=O)N/N=C/C(C=CC=C1O)=C1

MW: molecular weight, g/mol
LogSW: predicted aqueous solubility
RB: # of rotatable bonds
tPSA: total polar surface area (Angstroms^2)
hDon: # of hydrogen-bond donors
hAcc: # of hydrogen-bond acceptors
cLogP: predicted octanol-water partition coefficient (Chembridge algorithm)
molLogP: predicted octanol-water partition coefficient (Molsoft algorithm)
SMILES: Simplified Molecular Input Line Entry Specification

Compound # 13

Chembridge ID 5227326

MW 322.3

Formula C18 H14 N2 O4

Name 3,5-dihydroxy-N'-[(2-hydroxy-1-naphthyl)methylene]benzohydrazide

LogSW -2.849

RB 3

tPSA 102.15

hDon 4

hAcc 5

cLogP 2.2

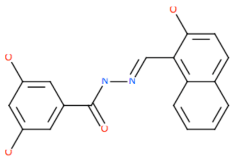

molLogP 3.61119

SMILES C(C(O)=CC(O)=C1)=C1C(=O)N/N=C/C(C(O)=CC1)=C2C=1C=CC=C2

Compound # 14

Chembridge ID 5252622

MW 352.3

Formula C16 H17 F N2 O6

Name N-[2-(acetylamino)-3-(4-fluorophenyl)acryloyl]glutamic acid

LogSW -3.028

RB 8

tPSA 132.8

hDon 4

hAcc 6

cLogP -.06

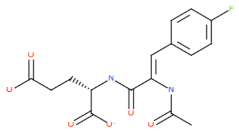

molLogP .451621

SMILES C(/C(C=CC(F)=C1)=C1)=C(/C(=O)N[C@H](C(=O)[O-])CCC(=O)[O-])NC(=O)C

Compound # 15

Chembridge ID 5256251

MW 298.3

Formula C14 H10 N4 O4

Name N-(6-anilino-4,7-dioxo-4,7-dihydro-2,1,3-benzoxadiazol-5-yl)acetamide

LogSW -2.17

RB 3

tPSA 114.19

hDon 2

hAcc 6

cLogP .93

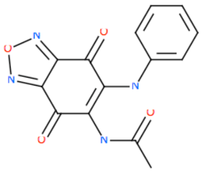

molLogP .926853

SMILES O=C(C(C(=NON1)C=1C(=O)C1NC(C=CC=C2)=C2)C=1NC(=O)C

Compound # 16

Chembridge ID 5256921

MW 301.3

Formula C14 H11 N3 O5

Name 4-hydroxy-N'-(2-hydroxy-5-nitrobenzylidene)benzohydrazide

LogSW -2.5

RB 3

tPSA 125.06

hDon 3

hAcc 6

cLogP 1.97

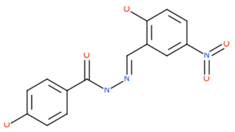

molLogP 2.21263

SMILES C(C(=C(O)C=C1)/C=N/NC(=O)C(C=CC(=C2)O)=C2)=C1N(=O)=O

MW: molecular weight, g/mol	hDon: # of hydrogen-bond donors
LogSW: predicted aqueous solubility	hAcc: # of hydrogen-bond acceptors
RB: # of rotatable bonds	cLogP: predicted octanol-water partition coefficient (Chembridge algorithm)
tPSA: total polar surface area (Angstroms^2)	molLogP: predicted octanol-water partition coefficient (Molsoft algorithm)
	SMILES: Simplified Molecular Input Line Entry Specification

Compound # 17

Chembridge ID 5264148

MW 272.3

Formula C14 H12 N2 O4

Name 3,5-dihydroxy-N'-(2-hydroxybenzylidene)benzohydrazide

LogSW -1.486

RB 3

tPSA 102.15

hDon 4

hAcc 5

cLogP 1.03

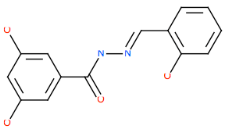

molLogP 2.28373

SMILES C(C(O)=CC(O)=C1)=C1C(=O)N/N=C/C(C=CC=C1)=C1O

Compound # 18

Chembridge ID 5266986

MW 272.3

Formula C14 H12 N2 O4

Name 3,4-dihydroxy-N'-(2-hydroxybenzylidene)benzohydrazide

LogSW -1.545

RB 3

tPSA 102.15

hDon 4

hAcc 5

cLogP 1.1

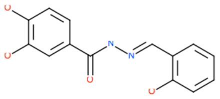

molLogP 2.16371

SMILES C(C(O)=C(O)C=C1)=C1C(=O)N/N=C/C(C=CC=C1)=C1O

Compound # 19

Chembridge ID 5267209

MW 317.3

Formula C14 H11 N3 O6

Name 3,4-dihydroxy-N'-(2-hydroxy-5-nitrobenzylidene)benzohydrazide

LogSW -2.189

RB 3

tPSA 145.29

hDon 4

hAcc 7

cLogP 1.47

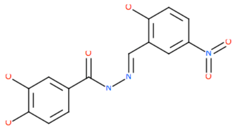

molLogP 1.83319

SMILES C(C(=C(O)C=C1)/C=N/NC(=O)C(C=CC(=C2O)O)=C2)=C1N(=O)=O

Compound # 20

Chembridge ID 5267603

MW 335.7

Formula C14 H10 Cl N3 O5

Name N'-(4-chloro-3-nitrobenzylidene)-3,4-dihydroxybenzohydrazide

LogSW -3.183

RB 3

tPSA 125.06

hDon 3

hAcc 6

cLogP 2.02

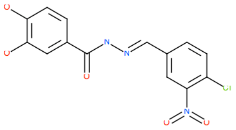

molLogP 2.80693

SMILES C(C(O)=C(C=C1)O)=C1C(=O)N/N=C/C(C=CC([Cl])=C1N(=O)=O)=C1

MW: molecular weight, g/mol
LogSW: predicted aqueous solubility
RB: # of rotatable bonds
tPSA: total polar surface area (Angstroms^2)
hDon: # of hydrogen-bond donors
hAcc: # of hydrogen-bond acceptors
cLogP: predicted octanol-water partition coefficient (Chembridge algorithm)
molLogP: predicted octanol-water partition coefficient (Molsoft algorithm)
SMILES: Simplified Molecular Input Line Entry Specification

Compound # 21

Chembridge ID 5280090

**MW**	288.3
**Formula**	C14 H12 N2 O5
**Name**	N'-(2,3-dihydroxybenzylidene)-3,5-dihydroxybenzohydrazide
**LogSW**	-1.09
**RB**	3
**tPSA**	122.38
**hDon**	5
**hAcc**	6
**cLogP**	0.43
**molLogP**	1.90429
**SMILES**	C(C(O)=CC(O)=C1)=C1C(=O)N/N=C/C(C=CC1)=C(C=1O)O

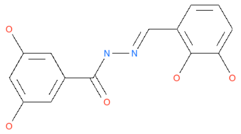

Compound # 22

Chembridge ID 5302171

**MW**	209.3
**Formula**	C9 H11 N3 O S
**Name**	2-(2-methylbenzoyl)hydrazinecarbothioamide
**LogSW**	-2.786
**RB**	2
**tPSA**	67.15
**hDon**	3
**hAcc**	1
**cLogP**	2.41
**molLogP**	.739915
**SMILES**	S=C(N)NNC(=O)C(C=CC=C1)=C1C

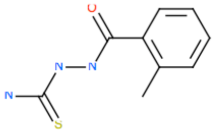

Compound # 23

Chembridge ID 5312165

**MW**	301.3
**Formula**	C14 H11 N3 O5
**Name**	3,4-dihydroxy-N'-(2-nitrobenzylidene)benzohydrazide
**LogSW**	-2.497
**RB**	3
**tPSA**	125.06
**hDon**	3
**hAcc**	6
**cLogP**	1.51
**molLogP**	2.09262
**SMILES**	C(C(O)=C(O)C=C1)=C1C(=O)N/N=C/C(C=CC=C1)=C1N(=O)=O

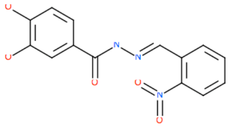

Compound # 24

Chembridge ID 5315106

**MW**	325.2
**Formula**	C14 H10 Cl2 N2 O3
**Name**	N'-(2,4-dichlorobenzylidene)-3,4-dihydroxybenzohydrazide
**LogSW**	-3.715
**RB**	3
**tPSA**	81.92
**hDon**	3
**hAcc**	4
**cLogP**	3.19
**molLogP**	3.85176
**SMILES**	C(C(O)=C(C=C1)O)=C1C(=O)N/N=C/C(C=CC([Cl])=C1)=C1[Cl]

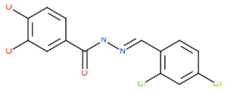

MW: molecular weight, g/mol	hDon: # of hydrogen-bond donors
LogSW: predicted aqueous solubility	hAcc: # of hydrogen-bond acceptors
RB: # of rotatable bonds	cLogP: predicted octanol-water partition coefficient (Chembridge algorithm)
tPSA: total polar surface area (Angstroms^2)	molLogP: predicted octanol-water partition coefficient (Molsoft algorithm)
	SMILES: Simplified Molecular Input Line Entry Specification

Compound # 25

Chembridge ID 5316181

**MW**	270.3
**Formula**	C15 H14 N2 O3
**Name**	3,4-dihydroxy-N'-(2-methylbenzylidene) benzohydrazide
**LogSW**	-2.522
**RB**	3
**tPSA**	81.92
**hDon**	3
**hAcc**	4
**cLogP**	2.26
**molLogP**	2.82429
**SMILES**	C(C(O)=C(O)C=C1)=C1C(=O)N/N=C/C(C=CC=C1)=C1C

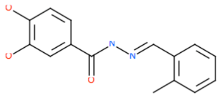

Compound # 26

Chembridge ID 5318411

**MW**	295.3
**Formula**	C16 H13 N3 O3
**Name**	3,4-dihydroxy-N'-(1H-indol-3-ylmethylene)benzohydrazide
**LogSW**	-2.268
**RB**	3
**tPSA**	97.71
**hDon**	4
**hAcc**	4
**cLogP**	1.75
**molLogP**	2.76061
**SMILES**	N(C(C=CC=C1)=C1C1/C=N/NC(=O)C(C=CC(O)=C2O)=C2)C=1

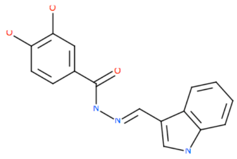

Compound # 27

Chembridge ID 5320663

**MW**	301.3
**Formula**	C14 H11 N3 O5
**Name**	2,4-dihydroxy-N'-(2-nitrobenzylidene) benzohydrazide
**LogSW**	-3.248
**RB**	3
**tPSA**	125.06
**hDon**	3
**hAcc**	6
**cLogP**	2.39
**molLogP**	2.09262
**SMILES**	C(C=C(O)C1)=C(C=1O)C(=O)N/N=C/C(C=CC=C1)=C1N(=O)=O

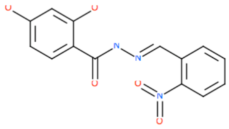

Compound # 28

Chembridge ID 5321784

**MW**	295.3
**Formula**	C16 H13 N3 O3
**Name**	3,5-dihydroxy-N'-(1H-indol-3-ylmethylene)benzohydrazide
**LogSW**	-2.209
**RB**	3
**tPSA**	97.71
**hDon**	4
**hAcc**	4
**cLogP**	1.68
**molLogP**	2.88063
**SMILES**	N(C(C=CC=C1)=C1C1/C=N/NC(=O)C(C=C(O)C=C2O)=C2)C=1

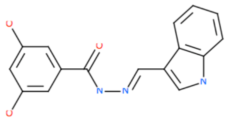

MW: molecular weight, g/mol
LogSW: predicted aqueous solubility
RB: # of rotatable bonds
tPSA: total polar surface area (Angstroms^2)
hDon: # of hydrogen-bond donors
hAcc: # of hydrogen-bond acceptors
cLogP: predicted octanol-water partition coefficient (Chembridge algorithm)
molLogP: predicted octanol-water partition coefficient (Molsoft algorithm)
SMILES: Simplified Molecular Input Line Entry Specification

Compound # 29

Chembridge ID 5322358

**MW**	301.3
**Formula**	C14 H11 N3 O5
**Name**	3,5-dihydroxy-N'-(2-nitrobenzylidene) benzohydrazide
**LogSW**	-2.437
**RB**	3
**tPSA**	125.06
**hDon**	3
**hAcc**	6
**cLogP**	1.44
**molLogP**	2.21263
**SMILES**	O=N(=O)C(C=CC=C1)=C1/C=N/NC(=O)C(C=C(O)C=C1O)=C1

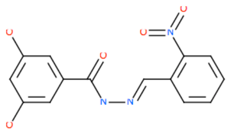

Compound # 30

Chembridge ID 5331708

**MW**	258.3
**Formula**	C14 H11 F N2 O2
**Name**	N'-(2-fluorobenzylidene)-4-hydroxybenzohydrazide
**LogSW**	-2.554
**RB**	3
**tPSA**	61.69
**hDon**	2
**hAcc**	3
**cLogP**	2.4
**molLogP**	3.07193
**SMILES**	C(=N/NC(=O)C(C=CC(O)=C1)=C1)/C(C=CC=C1)=C1F

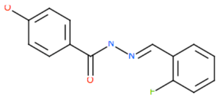

Compound # 31

Chembridge ID 5336667

**MW**	241.2
**Formula**	C13 H11 N3 O2
**Name**	4-hydroxy-N'-(3-pyridinylmethylene) benzohydrazide
**LogSW**	.271
**RB**	3
**tPSA**	74.58
**hDon**	2
**hAcc**	4
**cLogP**	.76
**molLogP**	1.87335
**SMILES**	C(=N/NC(=O)C(C=CC(O)=C1)=C1)/C(C=CC=N1)=C1

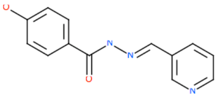

Compound # 32

Chembridge ID 5377378

**MW**	192.2
**Formula**	C9 H12 N4 O
**Name**	2-hydrazino-4-(methoxymethyl)-6-methylnicotinonitrile
**LogSW**	-1.594
**RB**	2
**tPSA**	83.96
**hDon**	2
**hAcc**	3
**cLogP**	.85
**molLogP**	-.071091
**SMILES**	N#CC(C(=CC(C)=N1)COC)=C1NN

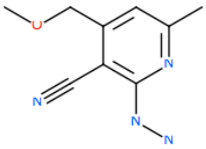

MW: molecular weight, g/mol	hDon: # of hydrogen-bond donors
LogSW: predicted aqueous solubility	hAcc: # of hydrogen-bond acceptors
RB: # of rotatable bonds	cLogP: predicted octanol-water partition coefficient (Chembridge algorithm)
tPSA: total polar surface area (Angstroms^2)	molLogP: predicted octanol-water partition coefficient (Molsoft algorithm)
	SMILES: Simplified Molecular Input Line Entry Specification

Compound # 33

Chembridge ID 5466567

**MW**	309.3
**Formula**	C17 H15 N3 O3
**Name**	4-hydroxy-N'-(1H-indol-3-ylmethylene)-3-methoxybenzohydrazide
**LogSW**	-3.215
**RB**	3
**tPSA**	86.71
**hDon**	3
**hAcc**	4
**cLogP**	2.06
**molLogP**	3.11008
**SMILES**	N(C(C=CC=C1)=C1C1/C=N/NC(=O)C(C=CC(O)=C2OC)=C2)C=1

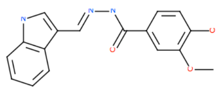

Compound # 34

Chembridge ID 5466778

**MW**	314.3
**Formula**	C16 H14 N2 O5
**Name**	N'-(1,3-benzodioxol-5-ylmethylene)-4-hydroxy-3-methoxybenzohydrazide
**LogSW**	-2.884
**RB**	3
**tPSA**	89.38
**hDon**	2
**hAcc**	6
**cLogP**	1.63
**molLogP**	3.04285
**SMILES**	C(C(=C(O)C=C1)OC)=C1C(=O)N/N=C/C(C=CC(OCO1)=C12)=C2

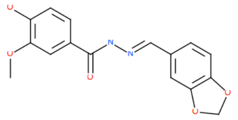

Compound # 35

Chembridge ID 5475747

**MW**	292.7
**Formula**	C14 H10 Cl F N2 O2
**Name**	N'-(2-chloro-6-fluorobenzylidene)-4-hydroxybenzohydrazide
**LogSW**	-3.411
**RB**	3
**tPSA**	61.69
**hDon**	2
**hAcc**	3
**cLogP**	3.11
**molLogP**	3.66622
**SMILES**	C(=N/NC(=O)C(C=CC(O)=C1)=C1)/C(C([Cl])=CC=C1)=C1F

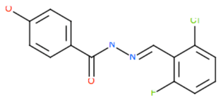

Compound # 36

Chembridge ID 5493440

**MW**	219.2
**Formula**	C11 H13 N3 O2
**Name**	N'-2-buten-1-ylidene-1-methyl-6-oxo-1,6-dihydro-3-pyridinecarbohydrazide
**LogSW**	-2.081
**RB**	3
**tPSA**	63.46
**hDon**	1
**hAcc**	3
**cLogP**	1.5
**molLogP**	.792339
**SMILES**	C(N(C)C(=O)C=C1)=C1C(=O)N/N=C/C=C/C

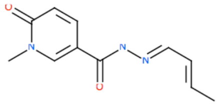

MW: molecular weight, g/mol	hDon: # of hydrogen-bond donors
LogSW: predicted aqueous solubility	hAcc: # of hydrogen-bond acceptors
RB: # of rotatable bonds	cLogP: predicted octanol-water partition coefficient (Chembridge algorithm)
tPSA: total polar surface area (Angstroms^2)	molLogP: predicted octanol-water partition coefficient (Molsoft algorithm)
	SMILES: Simplified Molecular Input Line Entry Specification

Compound # 37

Chembridge ID 5511358

MW 154.2
Formula C6 H10 N4 O
Name 2-hydrazino-4-methoxy-6-methylpyrimidine

LogSW -1.266
RB 1
tPSA 73.06
hDon 2
hAcc 3
cLogP 1.1

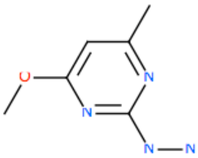

molLogP .043357

SMILES C(C(C)=NC(NN)=N1)=C1OC

Compound # 38

Chembridge ID 5521698

MW 262.3
Formula C12 H10 N2 O3 S
Name 2,4-dihydroxy-N'-(2-thienylmethylene) benzohydrazide

LogSW -2.489
RB 3
tPSA 81.92
hDon 3
hAcc 4
cLogP 2.29

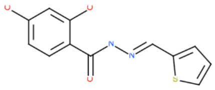

molLogP 2.4167

SMILES C(C=C(C1)O)=C(C=1O)C(=O)N/N=C/C(=CC=C1)S1

Compound # 39

Chembridge ID 5532286

MW 260.2
Formula C13 H12 N2 O4
Name N'-(2-furylmethylene)-4-hydroxy-3-methoxybenzohydrazide

LogSW -2.158
RB 3
tPSA 84.06
hDon 2
hAcc 5
cLogP 1.24

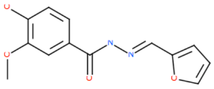

molLogP 2.11852

SMILES C(C(=C(O)C=C1)OC)=C1C(=O)N/N=C/C(=CC=C1)O1

Compound # 40

Chembridge ID 5562286

MW 238.2
Formula C10 H10 N2 O5
Name methyl N-(2-nitrobenzoyl)glycinate

LogSW -2.749
RB 3
tPSA 98.54
hDon 1
hAcc 5
cLogP .13

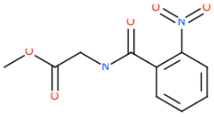

molLogP .333642

SMILES O=N(=O)C(C=CC=C1)=C1C(=O)NCC(=O)OC

MW: molecular weight, g/mol<br>LogSW: predicted aqueous solubility<br>RB: # of rotatable bonds<br>tPSA: total polar surface area (Angstroms^2)	hDon: # of hydrogen-bond donors<br>hAcc: # of hydrogen-bond acceptors<br>cLogP: predicted octanol-water partition coefficient (Chembridge algorithm)<br>molLogP: predicted octanol-water partition coefficient (Molsoft algorithm)<br>SMILES: Simplified Molecular Input Line Entry Specification

Compound # 41

Chembridge ID 5567417

MW 276.3
Formula C13 H12 N2 O3 S
Name 4-hydroxy-3-methoxy-N'-(2-thienylmethylene)benzohydrazide

LogSW -2.676
RB 3
tPSA 70.92
hDon 2
hAcc 4
cLogP 1.71

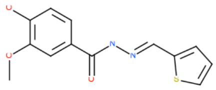

molLogP 2.76616

SMILES C(C(=C(O)C=C1)OC)=C1C(=O)N/N=C/C(=CC=C1)S1

Compound # 42

Chembridge ID 5630714

MW 320.7
Formula C15 H13 Cl N2 O4
Name N'-(5-chloro-2-hydroxybenzylidene)-4-hydroxy-3-methoxybenzohydrazide

LogSW -3.017
RB 3
tPSA 91.15
hDon 3
hAcc 5
cLogP 2.41

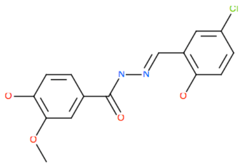

molLogP 3.2275

SMILES C(C(=C(O)C=C1)OC)=C1C(=O)N/N=C/C(C=C([Cl])C=C1)=C1O

Compound # 43

Chembridge ID 5774457

MW 267.3
Formula C16 H13 N O3
Name 4-(2-biphenylylamino)-4-oxo-2-butenoic acid

LogSW -2.73
RB 4
tPSA 66.4
hDon 2
hAcc 3
cLogP 1.85

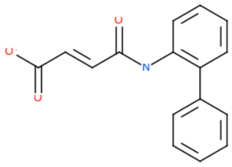

molLogP 3.25109

SMILES C(=C/C(=O)[O-])/C(=O)NC(C=CC=C1)=C1C(C=CC=C1)=C1

Compound # 44

Chembridge ID 5798683

MW 320.7
Formula C15 H13 Cl N2 O4
Name N'-[(2-chlorophenoxy)acetyl]-4-hydroxybenzohydrazide

LogSW -3
RB 5
tPSA 87.66
hDon 3
hAcc 4
cLogP 2.39

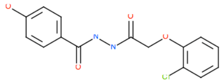

molLogP 2.13078

SMILES C(C(=O)NNC(=O)C(C=CC(O)=C1)=C1)OC(C=CC=C1)=C1[Cl]

MW: molecular weight, g/mol<br>LogSW: predicted aqueous solubility<br>RB: # of rotatable bonds<br>tPSA: total polar surface area (Angstroms^2)	hDon: # of hydrogen-bond donors<br>hAcc: # of hydrogen-bond acceptors<br>cLogP: predicted octanol-water partition coefficient (Chembridge algorithm)<br>molLogP: predicted octanol-water partition coefficient (Molsoft algorithm)<br>SMILES: Simplified Molecular Input Line Entry Specification

Compound # 45

Chembridge ID 5799663

MW 336.7
Formula C15 H13 Cl N2 O5
Name N'-[(2-chlorophenoxy)acetyl]-2,4-dihydroxybenzohydrazide

LogSW -3.441
RB 5
tPSA 107.89
hDon 4
hAcc 5
cLogP 2.77

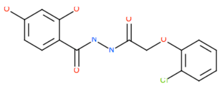

molLogP 1.75134

SMILES C(C=C(O)C1)=C(C=1O)C(=O)NNC(=O)COC(C=CC=C1)=C1[Cl]

Compound # 46

Chembridge ID 5851839

MW 275.2
Formula C10 H8 Cl2 N2 O S
Name 5-(2,5-dichlorobenzyl)-2-imino-1,3-thiazolidin-4-one

LogSW -4.478
RB 2
tPSA 52.95
hDon 2
hAcc 2
cLogP 3.83

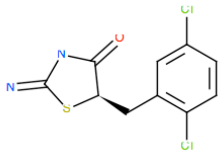

molLogP 2.44747

SMILES C([C@@H](SC(=N)N1)C1=O)C(C=C(C=C1)[Cl])=C1[Cl]

Compound # 47

Chembridge ID 5853551

MW 330.3
Formula C17 H18 N2 O5
Name N'-[(2,4-dimethylphenoxy)acetyl]-2,4-dihydroxybenzohydrazide

LogSW -3.719
RB 5
tPSA 107.89
hDon 4
hAcc 5
cLogP 3.15

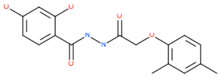

molLogP 1.83934

SMILES C(C=C(O)C1)=C(C=1O)C(=O)NNC(=O)COC(C=CC(=C1)C)=C1C

Compound # 48

Chembridge ID 6213095

MW 369.4
Formula C20 H23 N3 O4
Name 3-[(2,4-dihydroxybenzoyl)hydrazono]-N-mesitylbutanamide

LogSW -2.526
RB 5
tPSA 111.02
hDon 4
hAcc 5
cLogP 1.42

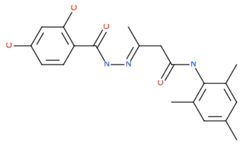

molLogP 3.0898

SMILES C(C(=O)NC(C(C)=CC(C)=C1)=C1C)/C(=N/NC(=O)C(C=CC(O)=C1)=C1O)C

MW: molecular weight, g/mol	hDon: # of hydrogen-bond donors
LogSW: predicted aqueous solubility	hAcc: # of hydrogen-bond acceptors
RB: # of rotatable bonds	cLogP: predicted octanol-water partition coefficient (Chembridge algorithm)
tPSA: total polar surface area (Angstroms^2)	molLogP: predicted octanol-water partition coefficient (Molsoft algorithm)
	SMILES: Simplified Molecular Input Line Entry Specification

Compound # 49

Chembridge ID 6326744

MW 336.4

Formula C15 H16 N2 O5 S

Name N-(2-hydroxyethyl)-2-[5-(4-methoxybenzylidene)-2,4-dioxo-1,3-thiazolidin-3-yl]acetamide

LogSW -2.549

RB 5

tPSA 95.94

hDon 2

hAcc 5

cLogP 1.05

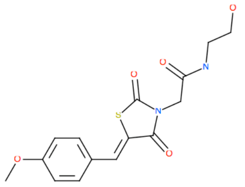

molLogP .945202

SMILES C(=C(S1)/C(N(C1=O)CC(=O)NCCO)=O)/C(C=CC(=C1)OC)=C1

Compound # 50

Chembridge ID 6348549

MW 366.4

Formula C16 H18 N2 O6 S

Name 2-[5-(3,4-dimethoxybenzylidene)-2,4-dioxo-1,3-thiazolidin-3-yl]-N-(2-hydroxyethyl)acetamide

LogSW -2.546

RB 5

tPSA 105.17

hDon 2

hAcc 6

cLogP .79

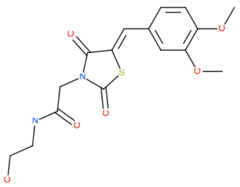

molLogP .915229

SMILES C(=C(S1)/C(N(C1=O)CC(=O)NCCO)=O)/C(C=CC(=C1OC)OC)=C1

Compound # 51

Chembridge ID 6356536

MW 306.3

Formula C14 H14 N2 O4 S

Name 2-(5-benzylidene-2,4-dioxo-1,3-thiazolidin-3-yl)-N-(2-hydroxyethyl) acetamide

LogSW -2.399

RB 5

tPSA 86.71

hDon 2

hAcc 4

cLogP 1.13

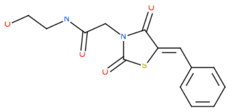

molLogP .855157

SMILES C(=C(S1)/C(N(C1=O)CC(=O)NCCO)=O)/C(C=CC=C1)=C1

Compound # 52

Chembridge ID 6631742

MW 422.4

Formula C16 H14 N4 O6 S2

Name N,N'-1,2-phenylenebis[2-(2,4-dioxo-1,3-thiazolidin-5-yl)acetamide]

LogSW -3.205

RB 6

tPSA 150.54

hDon 4

hAcc 6

cLogP -.45

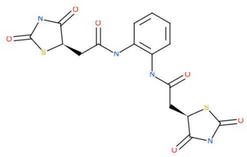

molLogP -.696903

SMILES C([C@@H](SC(=O)N1)C1=O)C(=O)NC(C=CC=C1)=C1NC(=O)C[C@@H](SC(=O)N1)C1=O

MW: molecular weight, g/mol
LogSW: predicted aqueous solubility
RB: # of rotatable bonds
tPSA: total polar surface area (Angstroms^2)
hDon: # of hydrogen-bond donors
hAcc: # of hydrogen-bond acceptors
cLogP: predicted octanol-water partition coefficient (Chembridge algorithm)
molLogP: predicted octanol-water partition coefficient (Molsoft algorithm)
SMILES: Simplified Molecular Input Line Entry Specification

Compound # 53

Chembridge ID 6946462

MW 362.4

Formula C21 H18 N2 O4

Name 3,3'-methylenebis(4-hydroxy-6-methyl-2(1H)-quinolinone)

LogSW -3.695

RB 2

tPSA 106.18

hDon 4

hAcc 4

cLogP 2.17

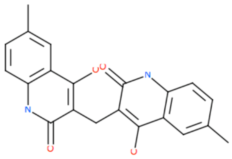

molLogP 3.08223

SMILES C(C(C(=O)N1)=C(C2=C1C=CC(C)=C2)O)C(C(=O)N1)=C(C2=C1C=CC(=C2)C)O

Compound # 54

Chembridge ID 6949907

MW 345.3

Formula C15 H11 N3 O5 S

Name 2-({[(2-nitrobenzoyl)amino]carbonothioyl}amino)benzoic acid

LogSW -4.089

RB 3

tPSA 121.57

hDon 3

hAcc 5

cLogP 2.32

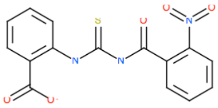

molLogP 1.98676

SMILES O=N(=O)C(C=CC=C1)=C1C(=O)NC(=S)NC(C=CC=C1)=C1C(=O)[O-]

Compound # 55

Chembridge ID 6988892

MW 342.4

Formula C18 H18 N2 O3 S

Name 3-({[(3,4-dimethylbenzoyl)amino]carbonothioyl}amino)-4-methylbenzoic acid

LogSW -4.711

RB 3

tPSA 78.43

hDon 3

hAcc 3

cLogP 3.53

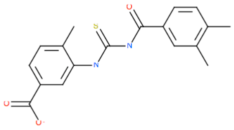

molLogP 3.52074

SMILES C(C(C(=O)[O-])=CC1)=C(C=1C)NC(=S)NC(=O)C(C=CC(=C1C)C)=C1

Compound # 56

Chembridge ID 7446357

MW 330.4

Formula C14 H14 N6 O2 S

Name 2-[(5-amino-7-oxo-7,8-dihydro[1,2,4]triazolo[4,3-a]pyrimidin-3-yl)thio]-N-(4-methylphenyl)acetamide

LogSW -3.189

RB 4

tPSA 118.17

hDon 3

hAcc 4

cLogP -.222

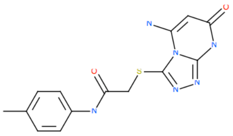

molLogP 1.3939

SMILES C(C(=O)N1)=C(N(C1=NN1)C=1SCC(=O)NC(C=CC(=C1)C)=C1)N

MW: molecular weight, g/mol<br>LogSW: predicted aqueous solubility<br>RB: # of rotatable bonds<br>tPSA: total polar surface area (Angstroms^2)	hDon: # of hydrogen-bond donors<br>hAcc: # of hydrogen-bond acceptors<br>cLogP: predicted octanol-water partition coefficient (Chembridge algorithm)<br>molLogP: predicted octanol-water partition coefficient (Molsoft algorithm)<br>SMILES: Simplified Molecular Input Line Entry Specification

Compound # 57

Chembridge ID 7507803

**MW**	338.1
**Formula**	C12 H8 Br N3 O4
**Name**	6-[2-(4-bromophenyl)vinyl]-4-hydroxy-5-nitro-2(1H)-pyrimidinone
**LogSW**	-3.035
**RB**	2
**tPSA**	109.12
**hDon**	2
**hAcc**	5
**cLogP**	2.003
**molLogP**	1.969
**SMILES**	C(/C(NC(=O)N1)=C(C=1O)N(=O)=O)=C/C(C=CC([Br])=C1)=C1

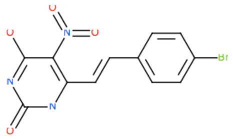

Compound # 58

Chembridge ID 7530375

**MW**	344.3
**Formula**	C16 H16 N4 O5
**Name**	4-hydroxy-6-{2-[4-(4-morpholinyl)phenyl]vinyl}-5-nitro-2(1H)-pyrimidinone
**LogSW**	-3.452
**RB**	3
**tPSA**	121.59
**hDon**	2
**hAcc**	6
**cLogP**	.96
**molLogP**	.975792
**SMILES**	C(/C(NC(=O)N1)=C(C=1O)N(=O)=O)=C/C(C=CC(=C1)N(CCOC2)C2)=C1

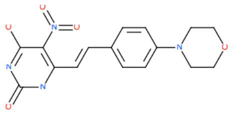

Compound # 59

Chembridge ID 7608933

**MW**	414.4
**Formula**	C24 H18 N2 O5
**Name**	4-{[(4-methylphenyl)amino]carbonyl}phenyl (1,3-dioxo-1,3-dihydro-2H-isoindol-2-yl)acetate
**LogSW**	-5.227
**RB**	6
**tPSA**	92.78
**hDon**	1
**hAcc**	5
**cLogP**	3.785
**molLogP**	3.94171
**SMILES**	C(C(=O)OC(C=CC(=C1)C(=O)NC(C=CC(C)=C2)=C2)=C1)N(C(=O)C(C=CC=C1)=C12)C2=O

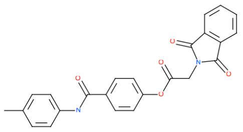

Compound # 60

Chembridge ID 7666888

**MW**	315.3
**Formula**	C15 H17 N5 O3
**Name**	methyl [2-({imino[(4-methylphenyl)amino]methyl}amino)-6-oxo-3,6-dihydro-4-pyrimidinyl]acetate
**LogSW**	-2.315
**RB**	6
**tPSA**	119.96
**hDon**	4
**hAcc**	5
**cLogP**	-.724
**molLogP**	.491161
**SMILES**	C(C(=O)N=C(NC(=N)NC(C=CC(C)=C1)=C1)N1)=C1CC(=O)OC

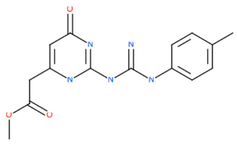

MW: molecular weight, g/mol
LogSW: predicted aqueous solubility
RB: # of rotatable bonds
tPSA: total polar surface area (Angstroms^2)
hDon: # of hydrogen-bond donors
hAcc: # of hydrogen-bond acceptors
cLogP: predicted octanol-water partition coefficient (Chembridge algorithm)
molLogP: predicted octanol-water partition coefficient (Molsoft algorithm)
SMILES: Simplified Molecular Input Line Entry Specification
