# Supplementary material for: Inhibition by stabilization: targeting the Plasmodium falciparum aldolase–TRAP complex
Source: Malar J. 2015 Aug 20;14:324. doi: 10.1186/s12936-015-0834-9 (PMC4545932; doi:10.1186/s12936-015-0834-9)

Additional file 5: Stereo figures of selected VLS hit poses. Stereo figures were prepared in the same orientation of some selected VLS hits docked to the coordinates of 2PC4 using the OpenEye software Fred. The surface rendering indicates the TRAP peptide in magenta, while the remaining of aldolase is kept grey, docked ligands are represented in sticks with the following color scheme: Compound 18 green, compound 24 cyan, compound 29 blue, compound 33 salmon and compound 42 yellow.

Compound 18,24,29,33 and 42 overlayed

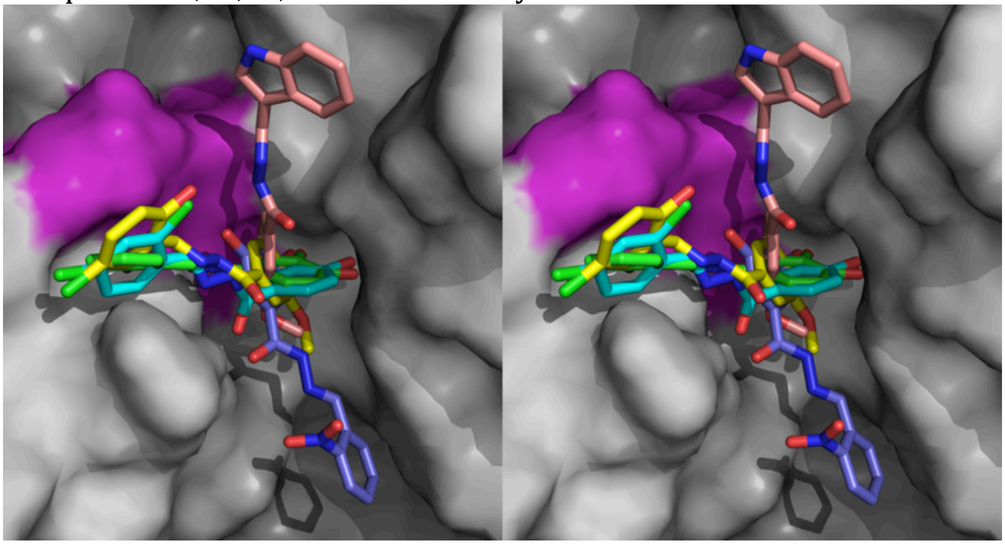

Compound 18

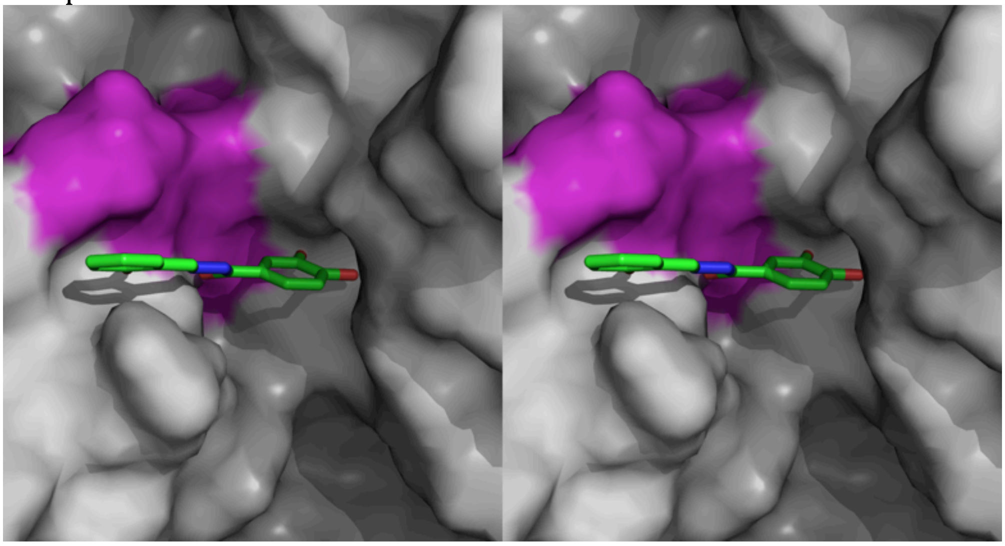

Compound 24

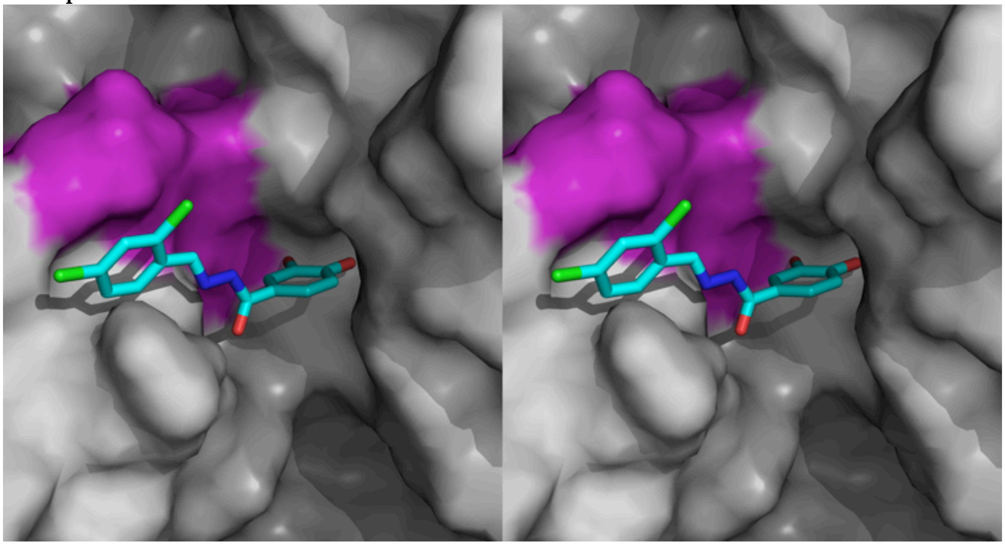

Compound 29

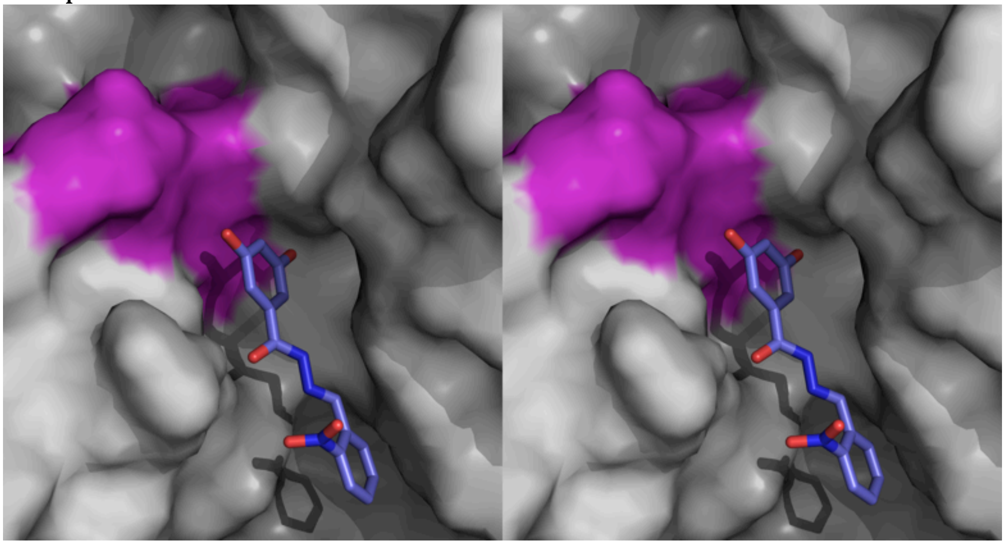

Compound 33

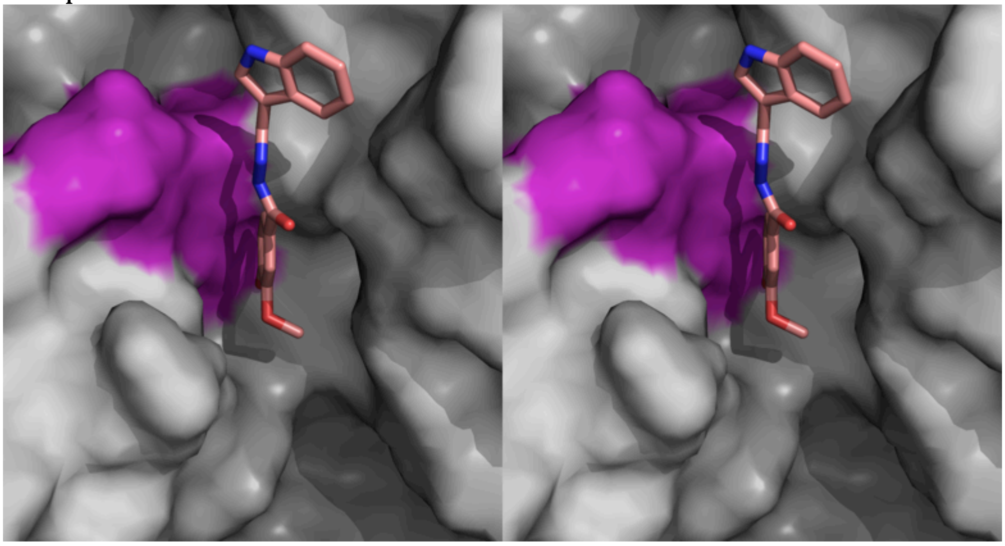

Compound 42

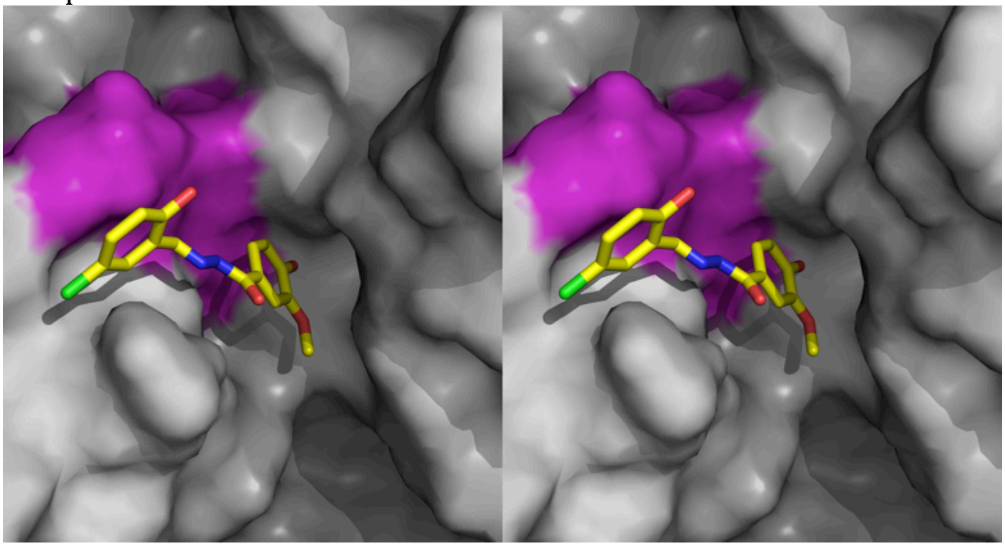

Supplement: Additional file 5. — Stereo figures of selected VLS hit poses. [file 12936_2015_834_MOESM5_ESM.pdf]
