# Supplementary material for: Inhibition by stabilization: targeting the Plasmodium falciparum aldolase–TRAP complex
Source: Malar J. 2015 Aug 20;14:324. doi: 10.1186/s12936-015-0834-9 (PMC4545932; doi:10.1186/s12936-015-0834-9)
Supplement: Additional file 6. — Sporozoite motility assay results for all compounds tested. [file 12936_2015_834_MOESM6_ESM.pdf]

Additional file 6:
Sporozoite motility assay results for all compounds tested
The effect on sporozoite motility was measured for a subset of the VLS hits.

Compound #	# of parasites counted	% motile cells	% with 1 trail	% with 2-10 trails	% with > 10 trails
DMSO	136	85	1	9	75
1	97	70	3	7	60
3	85	73	1	5	67
5	90	74	3	2	69
18	91	74	3	4	66
19	88	76	5	8	64
21	76	74	1	5	67
24	145	33	3	7	23
29	86	59	2	12	45
32	85	82	5	2	75
36	91	75	2	8	65
42	177	38	6	7	25
43	92	68	1	2	65

Sporozoite motility assay results for replicates of compounds 24 and 42
The motility assay was repeated in triplicate for compounds 24 and 42. The results are shown below.

Compound #	# of parasites counted	% motile cells	% with 1 trail	% with 2-10 trails	% with > 10 trails
DMSO (1)	124	82	2	10	70
DMSO (2)	106	83	4	9	70
DMSO (3)	115	78	2	8	69
DMSO Average	115	81	3	9	70
24 (1)	116	32	3	8	21
24 (2)	125	26	0	2	24
24 (3)	126	21	2	5	14
24 Average	122	26	2	5	20
42 (1)	129	46	7	9	30
42 (2)	128	53	5	14	34
42 (3)	121	50	5	17	28
42 Average	126	50	6	13	31
