# Supplementary material for: Inhibition by stabilization: targeting the Plasmodium falciparum aldolase–TRAP complex
Source: Malar J. 2015 Aug 20;14:324. doi: 10.1186/s12936-015-0834-9 (PMC4545932; doi:10.1186/s12936-015-0834-9)

Additional file 7: Ligplot representation of the TRAP peptides. Each TRAP peptide is represented with it's corresponding interacting residues in addition to hydrogen bonding distances.

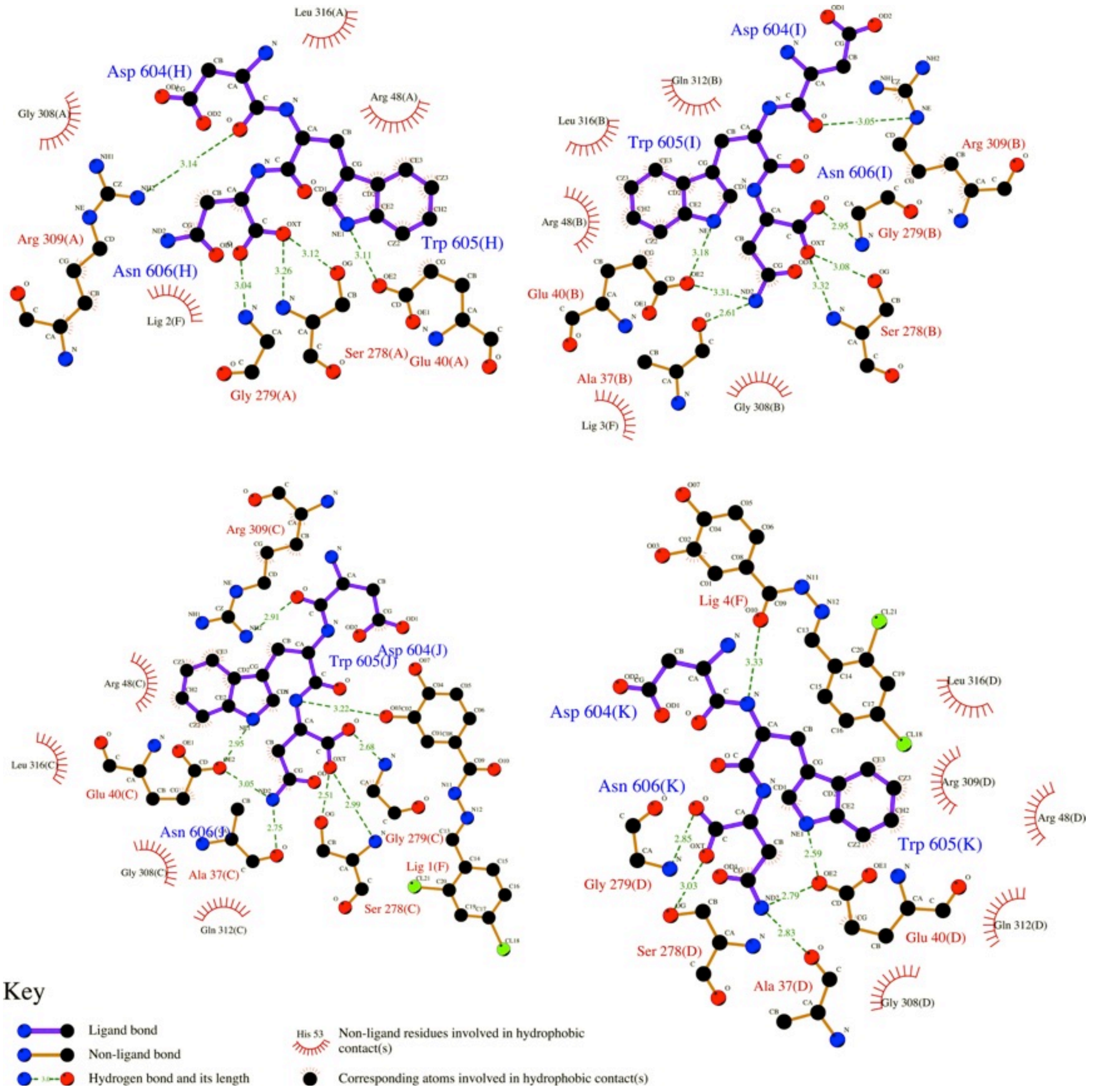

Supplement: Additional file 7. — Ligplot representation of the TRAP peptides. [file 12936_2015_834_MOESM7_ESM.pdf]
