# Supplementary material for: Inhibition by stabilization: targeting the Plasmodium falciparum aldolase–TRAP complex
Source: Malar J. 2015 Aug 20;14:324. doi: 10.1186/s12936-015-0834-9 (PMC4545932; doi:10.1186/s12936-015-0834-9)

Additional file 9: Stereo figures of each PfAldolase subunit bound to TRAP and C24. Stereo figures represent a close-up view of approximately the same orientation as depicted in Figure 5A of the main text. Each subunit is shown as ribbon representation with the 2Fo-Fc map contoured at 1.5s and the Fo-Fc difference density map at 3s levels.

A

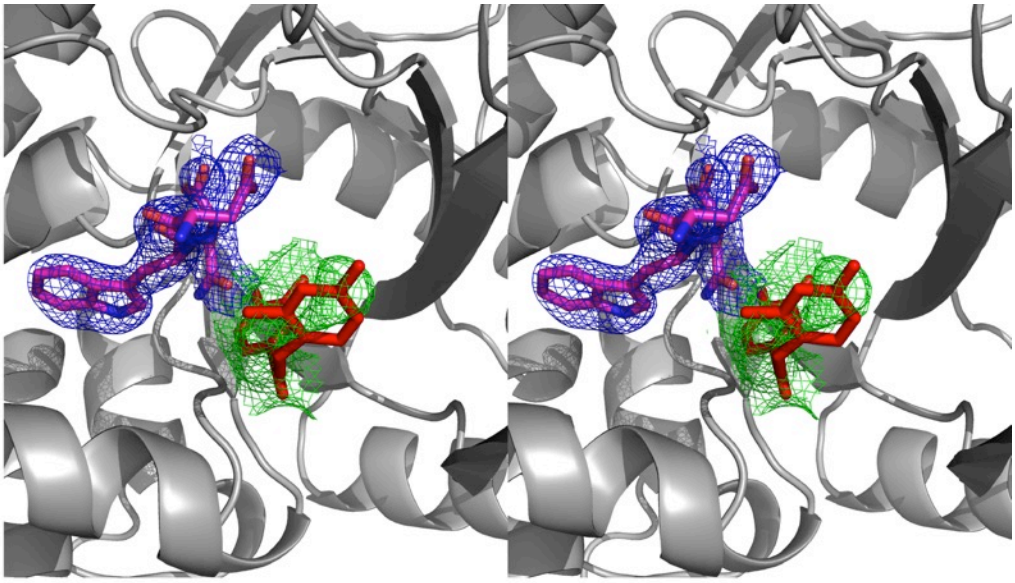

B

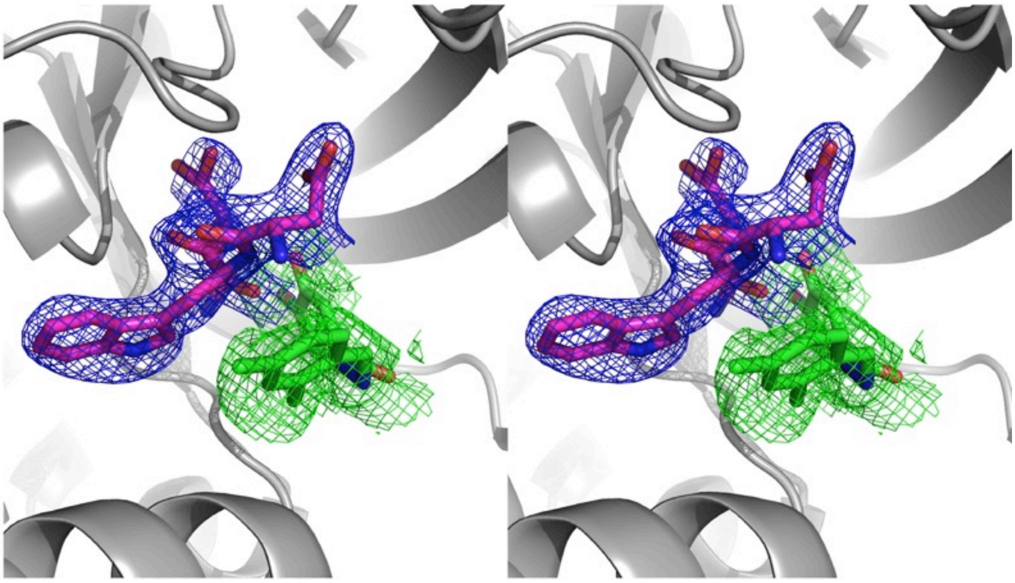

C

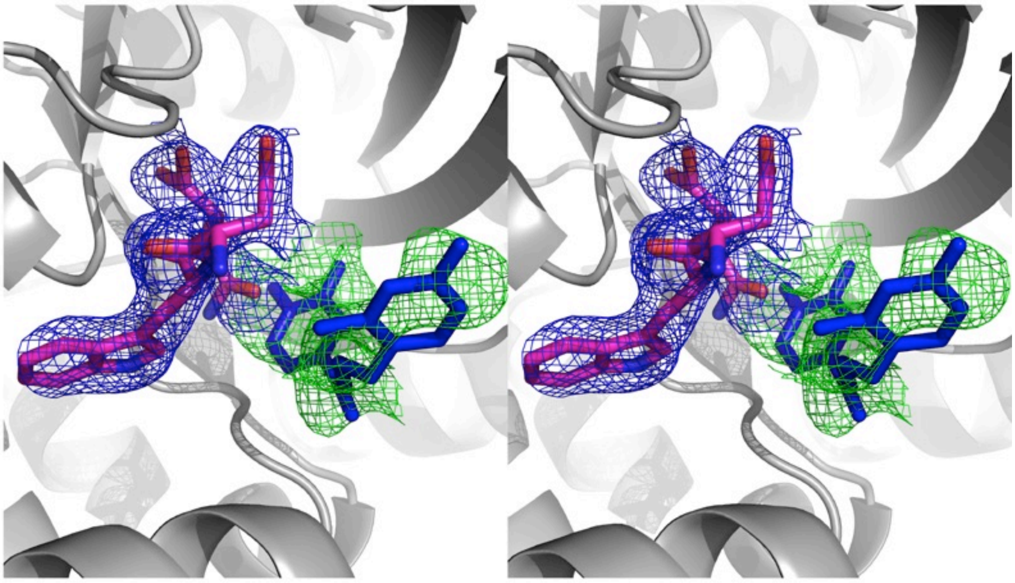

D

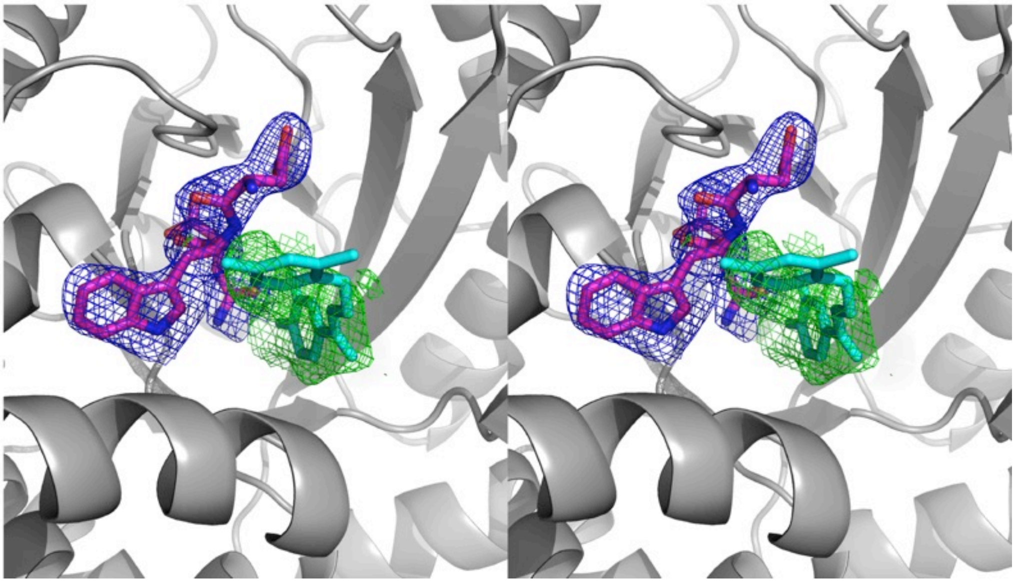

Supplement: Additional file 9. — Stereo figures of each PfAldolase subunit bound to TRAP and compound 24. [file 12936_2015_834_MOESM9_ESM.pdf]
