# Supplementary material for: Inhibition by stabilization: targeting the Plasmodium falciparum aldolase–TRAP complex
Source: Malar J. 2015 Aug 20;14:324. doi: 10.1186/s12936-015-0834-9 (PMC4545932; doi:10.1186/s12936-015-0834-9)

**Additional file 10: Stereo figure of Compound 24 in the crystal structure compared to the docking pose obtained with 2PC4.** A) Comparison between the docked pose (cyan sticks) and the observed orientation of compound 24 (orange sticks) in Chain A of the PfAldolase tetramer. The coordinates of 2PC4 are represented as ribbons and the corresponding TRAP-tail is shown in magenta sticks. The TRAP-tail of the ternary co-crystal structure 4TR9 is shown in orange sticks. B) Approximate rotation of 90 degrees towards the viewer compared to figure A.

A

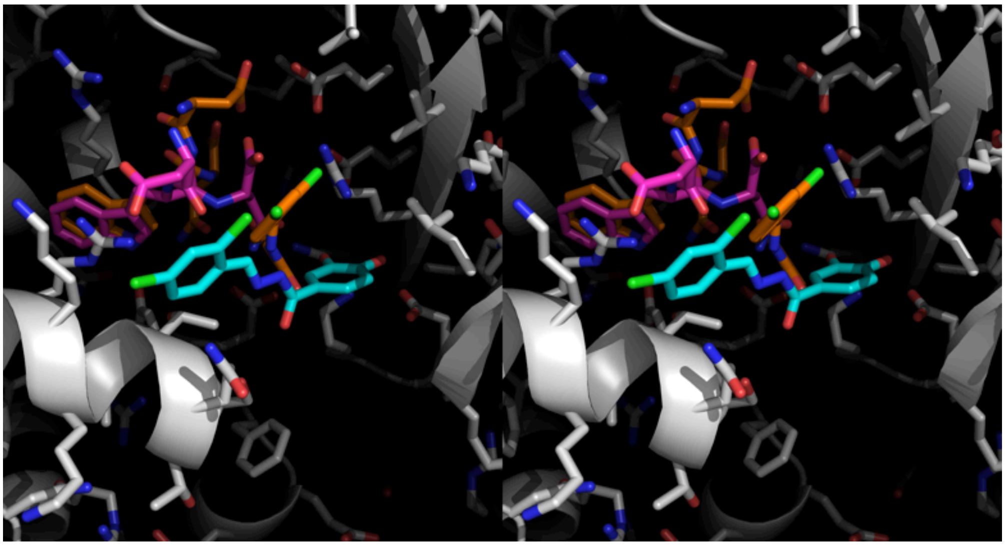

B

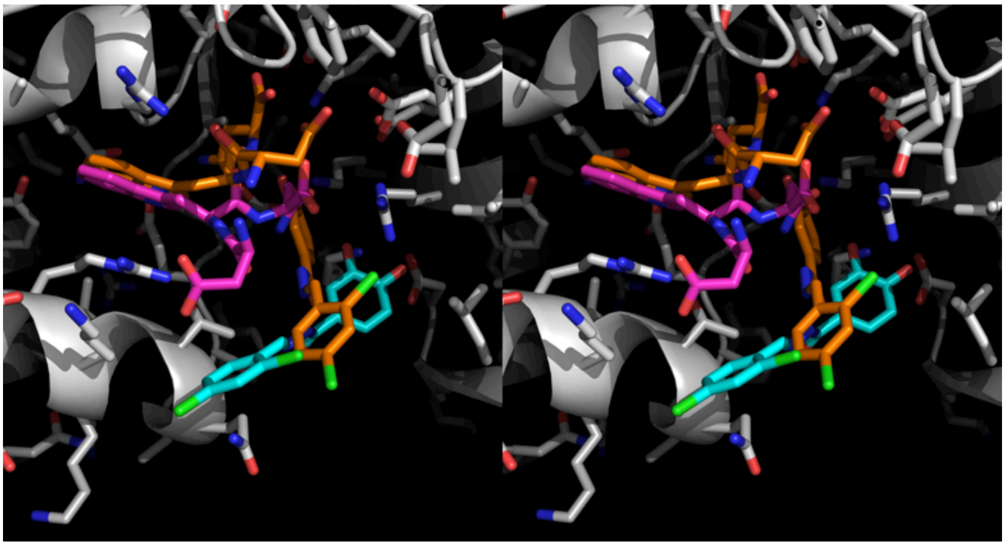

Supplement: Additional file 12. — Stereo figure of compound 24 in the crystal structure compared to the docking pose obtained with 2PC4. [file 12936_2015_834_MOESM12_ESM.pdf]
