# Supplementary material for: Inhibition by stabilization: targeting the Plasmodium falciparum aldolase–TRAP complex
Source: Malar J. 2015 Aug 20;14:324. doi: 10.1186/s12936-015-0834-9 (PMC4545932; doi:10.1186/s12936-015-0834-9)
Supplement: Additional file 13. — Identified C24 scaffold homologs within the TCAMs dataset. [file 12936_2015_834_MOESM13_ESM.pdf]

Additional File 11: Identified C24 scaffold homologs within the TCAMs dataset. The GSK TCAMS Dataset (hits from *P. falciparum* whole-cell screening) contains several active compounds with our identified scaffold as shown in Figure 4A. All 26784 active compounds from this dataset were structurally compared using ROCS from OpenEye Software, yielding several C24-homologs. The 25 closest hits based on shape and chemical similarity are shown below.

	Molecule	SOURCES
1	omega_27397_8 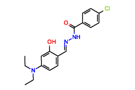	434141 (CHEMBL)
2	omega_32145_8 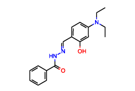	434086 (CHEMBL)
3	omega_31436_0 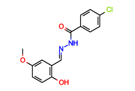	434396 (CHEMBL)
4	omega_33608_2 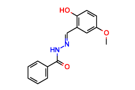	434506 (CHEMBL)
5	omega_27626_3 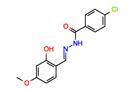	434271 (CHEMBL)

	Molecule	SOURCES
6	omega_28507_3 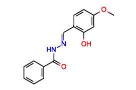	434229 (CHEMBL)
7	omega_22702_4 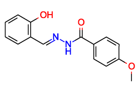	434119 (CHEMBL)
8	omega_36387_2 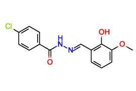	434258 (CHEMBL)
9	omega_24992_2 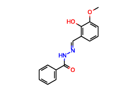	434110 (CHEMBL)
10	omega_40051_0 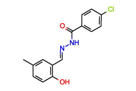	434517 (CHEMBL)

	Molecule	SOURCES
11	omega_29731_0 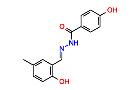	434464 (CHEMBL)
12	omega_35417_1 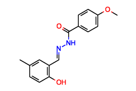	434524 (CHEMBL)
13	omega_32957_1 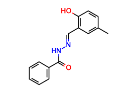	434279 (CHEMBL)
14	omega_31957_1 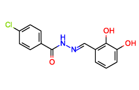	434108 (CHEMBL)
15	omega_27913_1 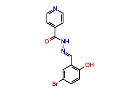	434424 (CHEMBL)

	Molecule	SOURCES
16	omega_26061_1 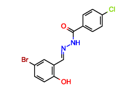	434136 (CHEMBL)
17	omega_30875_0 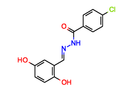	434196 (CHEMBL)
18	omega_30271_1 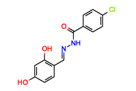	434038 (CHEMBL)
19	omega_22828_0 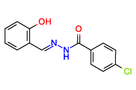	434118 (CHEMBL)
20	omega_23438_1 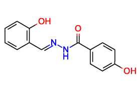	434117 (CHEMBL)

	Molecule	SOURCES
21	omega_29539_1 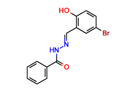	434432 (CHEMBL)
22	omega_33760_1 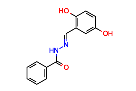	434495 (CHEMBL)
23	omega_30149_1 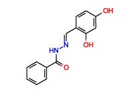	434183 (CHEMBL)
24	omega_23220_1 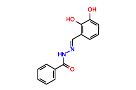	434096 (CHEMBL)
25	omega_30715_1 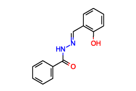	434103 (CHEMBL)
